# Mechanism and control strategies of asymmetric floor heave under dynamic pressure in roof-cutting gob-side entry retaining: a case study

**DOI:** 10.1038/s41598-026-58567-y

**Published:** 2026-06-22

**Authors:** Haohao Wang, Kunyu Liu, Zhujun Shao, Dongshan Yang, Shukun Zhang, Shuai Wang, Zhibiao Guo

**Affiliations:** 1 School of City and Architecture Engineering, Zaozhuang University, Zaozhuang, 277160 China; 2 Gaozhuang Coal Mine, ZaoZhuang Mining Group Co., Ltd, Weishan, 277605 Shandong China; 3 China University of Mining and Technology-Beijing, Beijing, 100083 China

**Keywords:** Roadways under dynamic pressure, Asymmetric floor heave, Inverted floor beam, Pressure-equalizing support, Energy science and technology, Engineering

## Abstract

Roof cutting for gob-side entry retaining actively modifies the structural configuration of the roof and the two ribs, which intensifies the inherent asymmetry in both the surrounding rock structure and the stress distribution. This structural evolution makes asymmetric floor heave under dynamic pressure a critical challenge. In this study, the mechanisms and control strategies for asymmetric floor heave in roadways under dynamic pressure were investigated through physical model tests, theoretical analysis, and field measurements, followed by a successful engineering application at the Zhaogu No. 1 Mine. Physical model tests verified the advantages of roof cutting in controlling surrounding rock deformation and revealed the evolutionary process of tensile strain in the roadway floor from shallow to deep strata. A mechanical model of asymmetric floor failure under mining-induced dynamic pressure in roof-cutting gob-side entry retaining was established, and the failure depth and plastic zone width after roof cutting were derived using Rankine’s earth pressure theory. Furthermore, a pressure-equalizing support scheme was proposed, incorporating roof pre-splitting, constant resistance & large-deformation (CRLD) support for the roof and coal ribs, and inverted floor beam support for the floor. Field results demonstrated that, compared to the unrestricted floor control scheme, this pressure-equalizing support scheme reduced floor heave by 46.1%. The effectiveness and feasibility of the proposed integrated control scheme were systematically verified through theoretical analysis, physical similarity simulation, and field validation.

## Introduction

 As coal mining extends into greater depths, large deformations in soft rock roadways—particularly severe floor heave—have become a critical bottleneck constraining the safe and efficient production of deep mines. Under the superposition of high in-situ stress and intense mining-induced stress, deep surrounding rock exhibits typical engineering soft rock characteristics, such as pronounced rheology and poor long-term stability^1–3^. In recent years, with the high-intensity extraction of coal seams under complex geological conditions, the phenomenon of floor heave instability has demonstrated a trend of increased frequency, greater magnitude of deformation, and intensified evolutionary processes. This not only significantly elevates roadway maintenance costs but also places more stringent demands on the prevention and control of dynamic disasters in deep mines^4–6^.

Regarding the underlying mechanisms of floor heave, extensive research has been conducted from various perspectives, including geological conditions, stress evolution, and hydro-mechanical coupling, yielding significant theoretical advancements. Chen et al.^7^ revealed the stress concentration effect at the arch foot caused by large-span flat sections and, combined with the sharp decline in mudstone bearing capacity under high hydraulic head pressure, clarified the synergistic floor heave mechanism driven by high seepage pressure and soft rock softening. Wu et al.^8^ focused on steeply inclined strata (60°) and proposed that under the influence of concentrated mining stress and high horizontal stress, roadway floor strata are prone to slip and shear along bedding planes, resulting in asymmetric floor heave. Through long-term field measurements, Chang et al.^9^ found that repeated moisture intrusion causes the water content of loess in tunnel floors to grow exponentially, driving a transition toward a rheological state and inducing upheaval. Wei et al.^10^ utilized 3DEC simulations to confirm that the coupling of deep high abutment pressure and dynamic loading from main roof breakage causes floor stress and displacement to exhibit an “inverted right-leaning V-shaped” asymmetric distribution toward the coal pillar. From the perspective of “symmetry breaking,” Qin et al.^11^ and Yang et al.^12^ noted that coal extraction disrupts the mechanical equilibrium of the pillar-roadway system, leading to stress concentration toward the pillar; their numerical results further established that floor heave is positively correlated with burial depth and advance speed, while being inversely correlated with pillar width. Li et al.^13^ utilized UDEC simulations and a simply-supported beam mechanical model to reveal that floor heave is primarily dominated by tensile failure, with the magnitude showing an exponential relationship with horizontal in-situ stress. Feng et al.^14^ pointed out that non-uniform abutment pressure from overlying remnant pillars and gobs leads to the deflection of principal stress directions in the underlying coal seam, where the non-uniform stress field induces asymmetric “butterfly-shaped failure” and the rapid expansion of the floor’s asymmetric plastic zone. Furthermore, Małkowski et al.^15^ elucidated the dynamic evolution of floor heave, wherein stress exceeding strength limits triggers crack connectivity and the formation of fractured zones; notably, under aqueous conditions, water infiltration significantly reduces the strength of clay minerals and triggers volumetric expansion.

To address floor heave under various working conditions, researchers have developed diversified control technologies ranging from active reinforcement and stress transfer to systematic synergy. He et al.^16^ developed a “double-sealing floor-reinforced inverted arch” scheme, which utilizes a “bolt + metal mesh + shotcrete” combination to form a fully enclosed system, achieving instantaneous sealing of floor water sources and an enhancement of support strength. Yu et al.^17^ innovatively proposed a collaborative control technology using “floor corner anchoring-grouting piles,” which significantly improves the overall bearing capacity of weakly cemented rock masses by filling fractures with grout, isolating moisture, and applying active constraint via anchors. Based on the “roof-rib-floor” collaborative support concept, Wang et al.^18^ proposed a combined scheme consisting of “floor leveling, anchor cable reinforcement, and concrete hardening.” Fu et al.^19^ guided stress transfer into the deep rock mass by excavating stress-relief slots at the roadway floor corners, combined with anchoring-grouting and waste-rock backfilling to enhance surrounding rock stability. For deep soft rock, He et al.^20^ proposed “differentiated” combined support, the core of which includes “bolt-mesh-cable-shotcrete + inverted floor arch + grouting,” with additional O-shaped closed steel frames installed in sections under intense dynamic pressure to resist extreme asymmetric deformation. Wen et al.^21^ presented a “modification reinforcement + collaborative support” strategy; by employing a 0.5 m thick inverted arch and reinforced floor corner bolts, the stress peak was transferred to the deep surrounding rock, effectively controlling shear failure. Furthermore, Zhang et al.^22^ designed a combined anchoring-grouting technology featuring “full-section anchor cables + prefabricated block inverted arches” based on the “Strong-Weak-Strong” composite bearing ring theory, which suppressed the extrusion effect of broken rock masses by increasing the flexural rigidity of the floor. Currently, floor-heave deformation is commonly monitored using either internal rock-mass strain measurements or surface strain monitoring techniques. Internal monitoring methods can provide more direct information on the deformation behavior of deep strata, whereas surface monitoring methods are capable of capturing the evolution and spatial distribution of strain over a relatively large area. In particular, full-field surface deformation monitoring techniques, such as Digital Image Correlation (DIC)^23–26^, can effectively identify the initiation and propagation regions of floor-heave deformation, thereby providing valuable information for understanding the development mechanism of floor instability.

In this study, the movement of overlying strata and the deformation patterns of surrounding rock in roadways formed by roof cutting were investigated through physical model tests. A Rankine-based mechanical model of asymmetric floor failure under mining-induced dynamic pressure in roof-cutting gob-side entry retaining was established., which further elucidated the evolutionary laws of floor heave. Subsequently, A coupled support strategy integrating roof pre-splitting, CRLD anchor cable reinforcement, and inverted floor beam support was proposed and successfully implemented to suppress the development of severe asymmetric floor heave. This research not only expands the theoretical scope of surrounding rock control for roof-cutting gob-side entry retaining but also provides an effective solution for mitigating asymmetric floor heave in deep soft rock roadways.

## Project overview

### Overview of working face

**Fig. 1:**
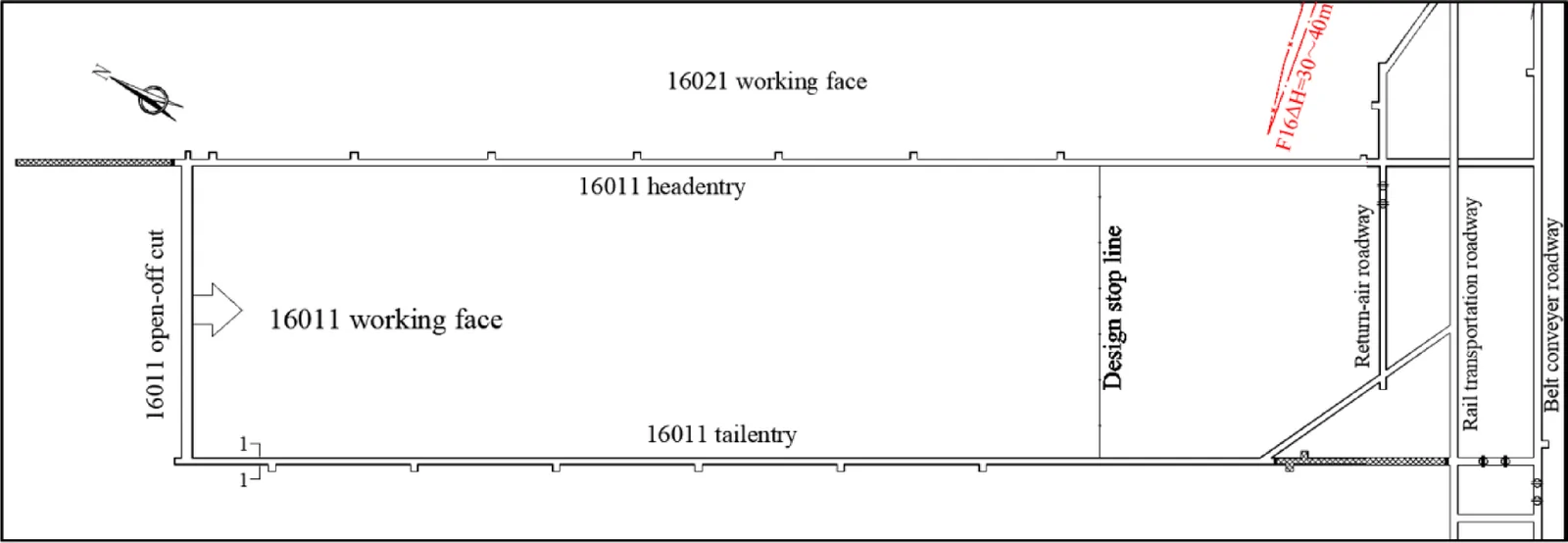
Layout of 16,011 working face.

The 16,011 working face of the Zhaogu No. 1 Mine is situated at the − 525 m level, featuring an average coal seam thickness of 5.8 m and a mean dip angle of 2.5°. As the inaugural working face of the West Six Panel, the 16,011 working face extends 205.5 m along the dip direction, with a designed advance length of 650 m. The lower gateway of this working face serves as the air return roadway and is designated as the experimental roadway for gob-side entry retaining by roof cutting, while the upper gateway functions as the transport roadway. Both gateways were developed within the No. 2 − 1 coal seam and excavated along its roof strata. The layout of the roadways is illustrated in Fig. 1.

### Rock character analysis of coal seam roof and floor

**Fig. 2:**
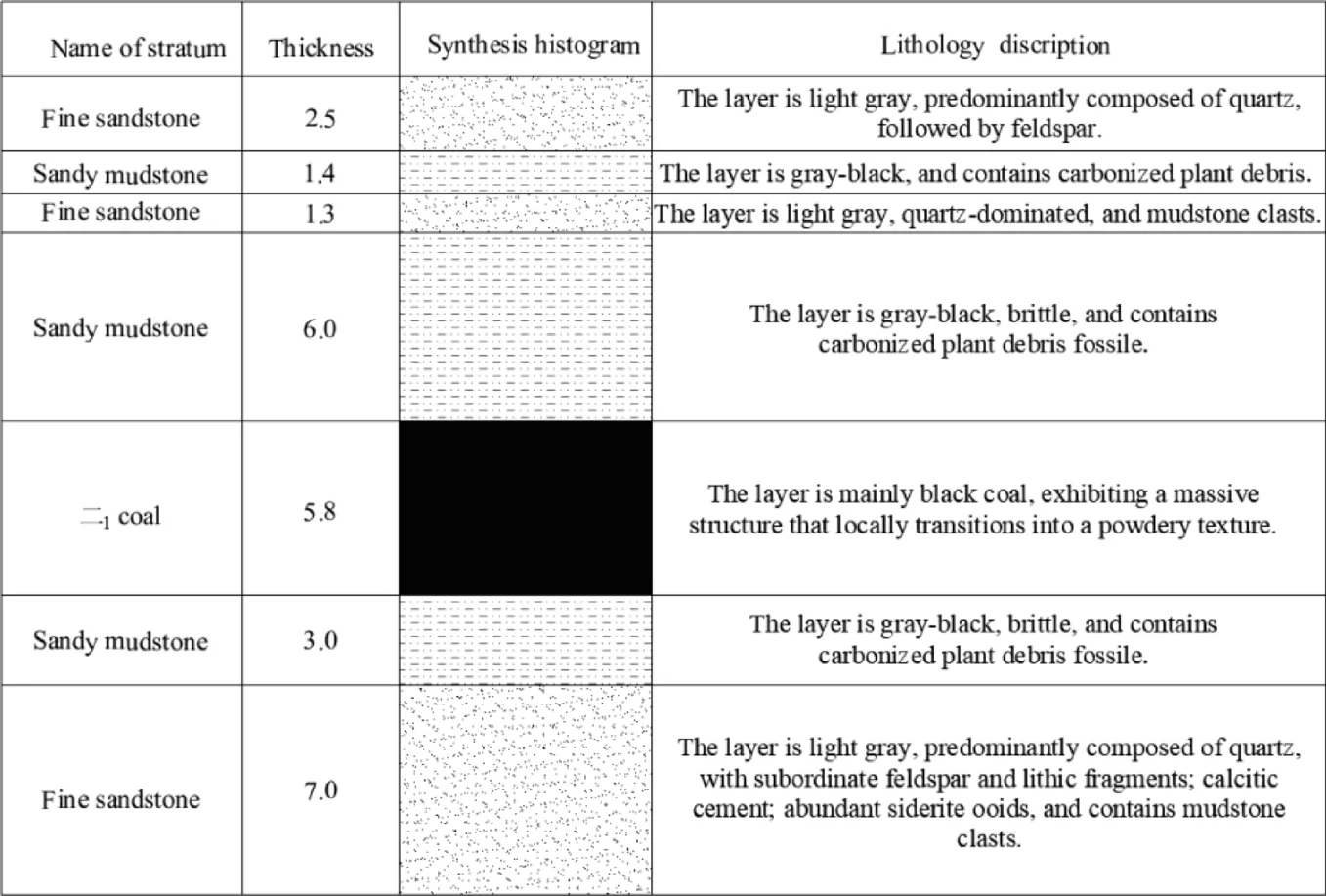
Distribution of the roof and floor strata of the coal seam.

The No. 2 − 1 coal seam and its roof and floor strata are primarily situated within the Shanxi Formation of the Lower Permian system, with an average burial depth of 560 m. The roof of the coal seam is characterized by interbedded layers of medium-grained sandstone and sandy mudstone; from the coal seam upward, the thicknesses of these layers are 6.0 m, 1.3 m, 1.4 m, and 2.5 m, respectively. Regarding the floor strata, there are 3.0 m of sandy mudstone and 7.0 m of medium-grained sandstone, sequenced from top to bottom. The specific rock stratum distribution of 16,011 working face is shown in Fig. 2.

The physical and mechanical parameters of the surrounding rock are shown in Table 1.

**Table 1:** Physical and mechanical parameters of surrounding rocks.

Rock type	Density (g/cm^3^)	Elastic modulus E (GPa)	Poisson’s ratio µ	Internal friction angle (°)	Cohesion (MPa)	Tensile strength (MPa)	UCS (MPa)
Sandy mudstone	2.55	4.61	0.26	32	2.24	1.23	37.06
二_1_ coal	1.33	2.17	0.31	24	0.81	0.55	17.15
Fine sandstone	2.43	3.90	0.24	30	2.47	1.46	54.82

### Design of roadway support system

**Fig. 3:**
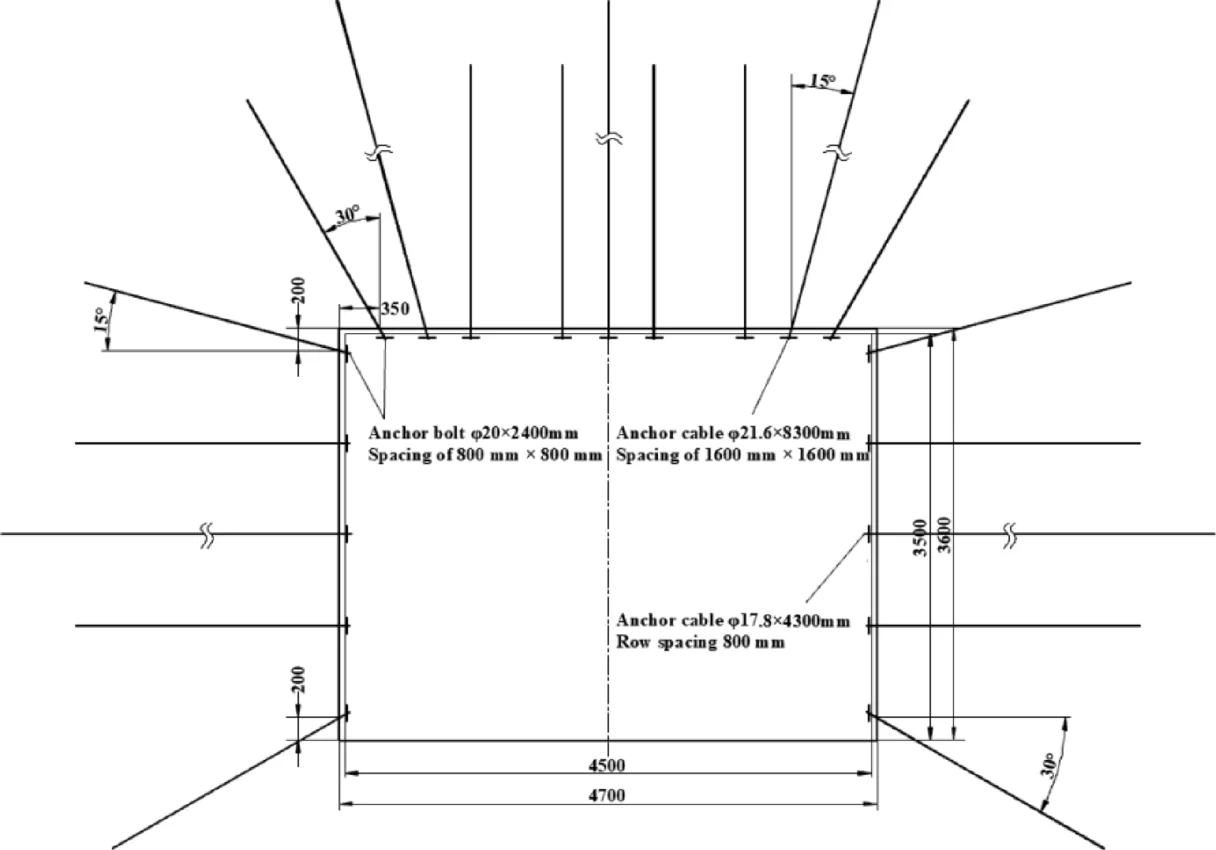
Cross-sectional view of initial support for mining roadway.

The air return gateway of the 16,011 working face features a net cross-section with dimensions of 4500 mm × 3500 mm (width × height). The initial support scheme adopts a combined bolt-and-cable support system. Specifically, the roof is reinforced with six rock bolts (Φ20 mm × 2400 mm) at a spacing and row spacing of 800 mm × 800 mm. Each of the two ribs is supported by five rock bolts, with the same specifications and spacing as those used in the roof. For the roof reinforcement, anchor cables (Φ21.6 mm × 8300 mm) are arranged with a spacing and row spacing of 1600 mm × 1600 mm. Additionally, a single anchor cable (Φ17.8 mm × 4300 mm) is installed at the center of each rib with a row spacing of 800 mm. The cross-sectional support design is illustrated in Fig. 3.

### Floor heave conditions of adjacent roadways

**Fig. 4:**
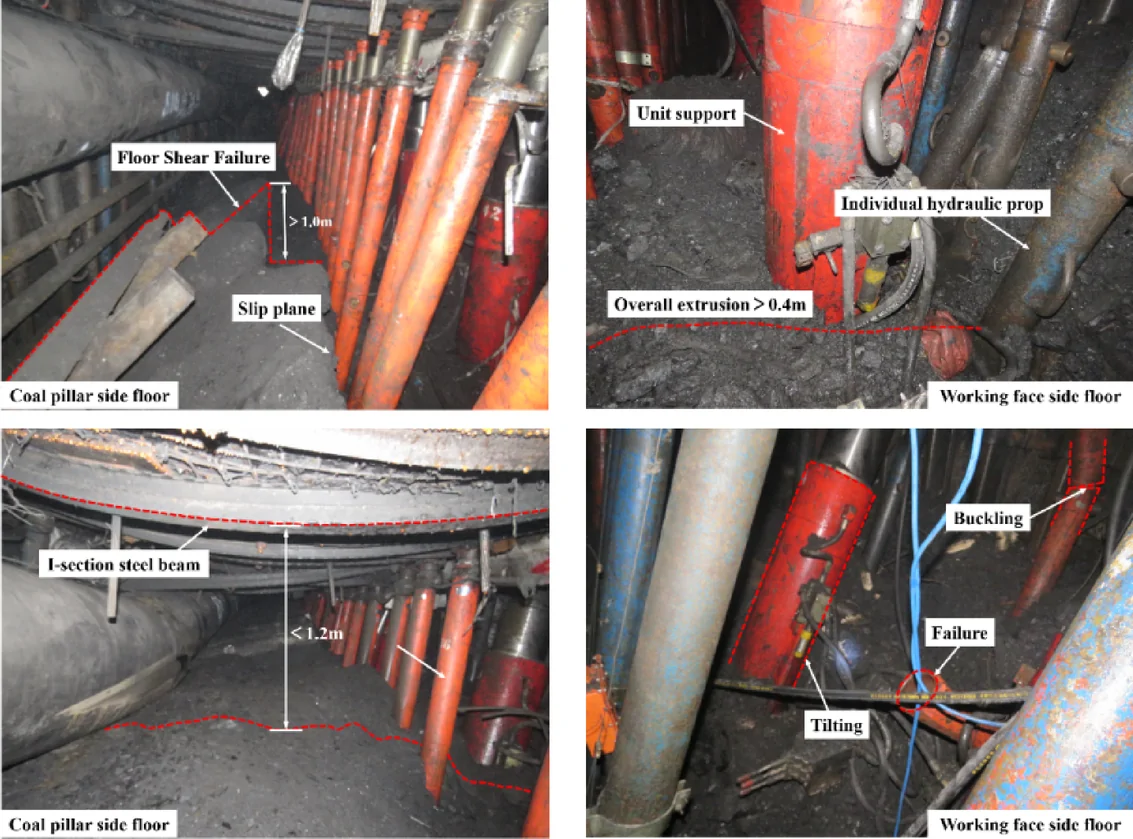
Field observations of floor heave in adjacent roadways.

Subjected to the persistent influence of abutment pressure during the extraction of the adjacent panel, the return gateroad of the 16,011 working face underwent intense floor heave within the coal seam. Shear failure occurred on the pillar side (track-laying side), resulting in “bench-like” bulging. In contrast, the face side exhibited “extrusion-type” heave under the high-intensity support of single props and unit supports. Such deformation culminated in an integral floor heave, which not only caused the roof-to-floor convergence to surpass 2500 mm but also led to large-scale failure of the support systems (Fig. 4).

### Research on the floor deformation mechanism of gob-side entry retaining by roof cutting

#### Physical similarity simulation of gob-side entry retaining by roof cutting

The simulation was conducted using the YDM-C two-dimensional physically similar simulation apparatus. The device has geometric dimensions of 1600 × 1600 × 400 mm and primarily consists of four components: the model frame, specimen molds, a hydraulic-driven control console, and a data monitoring and acquisition system. It is designed to meet the experimental requirements for underground coal mining and slope stability evaluation.

**Fig. 5:**
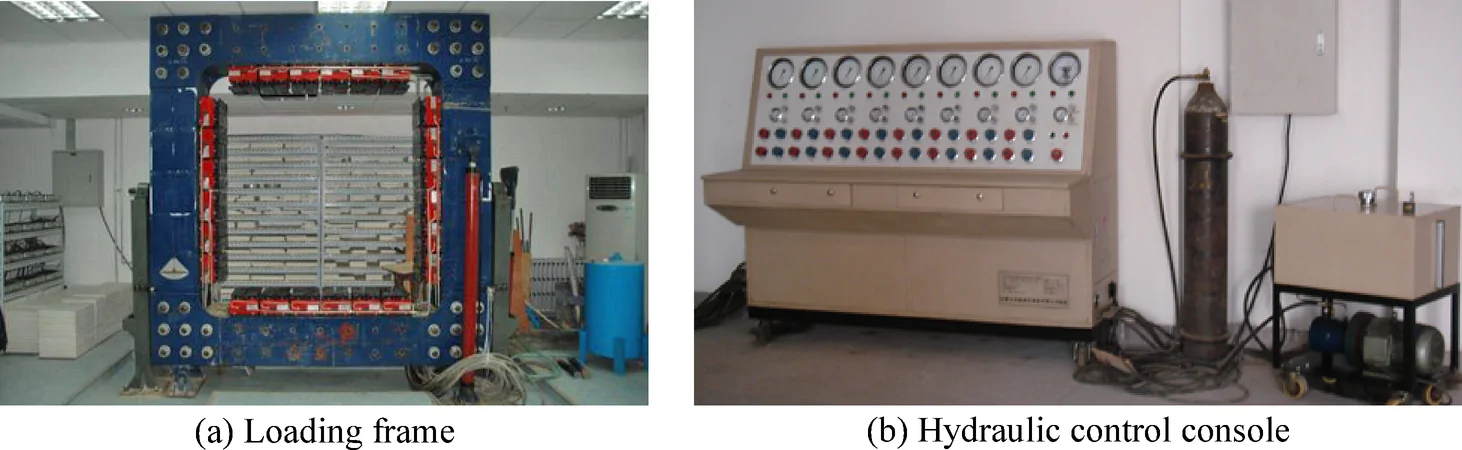
YDM-C material similarity simulation test device.

Six uniformly distributed loading actuators were arranged on each of the top, bottom, left, and right sides of the model frame. The actuators installed on the top and lateral boundaries could be independently controlled, allowing nonlinear loading paths to be applied and facilitating the simulation of the in-situ stress field and mining-induced stress evolution during roadway excavation and panel extraction. The maximum pressure applied via the control console is 5 MPa, with a control precision of 0.1 MPa. The model frame and hydraulic-driven console of the YDM-C apparatus are illustrated in Fig. 5.

**Fig. 6:**
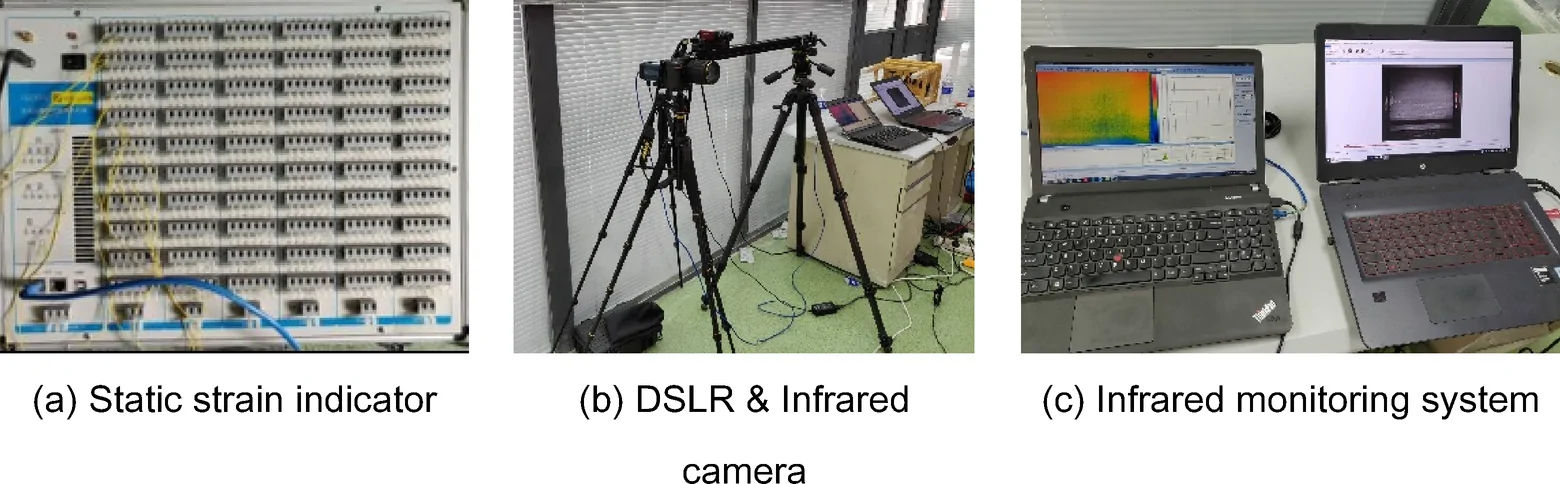
Monitoring equipment and processing system of similar material model.

The data acquisition system primarily consists of a YSV8360 static strain testing system, a Digital Single-Lens Reflex (DSLR) camera, and an infrared thermal imager, along with complementary data processing software, as illustrated in Fig. 6.

**Fig. 7:**
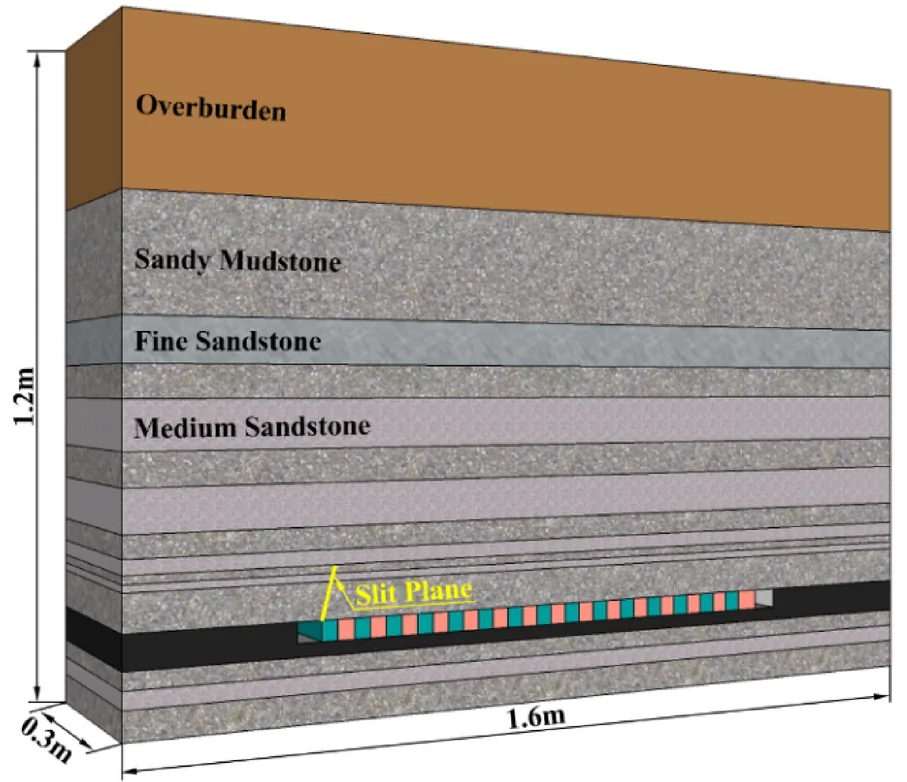
Schematic diagram of the physical similarity model design.

The actual dimensions of the roof-cut roadway are 4500 × 3500 mm, while the effective loading dimensions of the physical simulation system are 1600 × 1600 × 400 mm. Considering the proportional relationship between the stope area and the roadway size, a geometric similarity ratio (*C*_L_) of 1:100 was adopted. Accordingly, the physical model represents a field-scale rock mass of 160 × 160 × 40 m, with the roadway dimensions in the model scaled to 45 × 35 mm. In addition, the density similarity ratio (*C*r) of the model was determined to be 1.44:1. According to the similarity theory, the stress similarity ratio (C_σ_), strength similarity ratio (*C*_p_), and elastic modulus similarity ratio (*C*_E_) were all calculated as 144:1. The configuration of the physical model and its strata distribution are illustrated in Fig. 7.

Fine sand was used as the aggregate material, while lime and gypsum were employed as cementing agents in the physical similarity simulation. Considering the unique physical and mechanical properties of the coal seam, an appropriate amount of fly ash and sawdust was incorporated into the coal-seam simulation material. An orthogonal experimental design was conducted to determine the optimal material mix proportions and corresponding mechanical properties of each simulated stratum. The mix proportions of the materials used to simulate the five strata above and three strata below the coal seam are presented in Table 2.

**Table 2:** Similar material ratio and mechanical parameters.

Rcok stratum number	Thickness (m)	Lithology	Proportion number	Model thickness(mm)	Density (g/cm^3^)	Weight of water	Compressive strength (MPa)
9	3.8	Sandy mudstone	10:9:1	38	15.9	1/10	0.24
8	2.5	Fine sandstone	7:8:2	25	14.2	1/10	0.31
7	1.4	Sandy mudstone	10:9:1	17	15.9	1/10	0.21
6	1.3	Fine sandstone	7:8:2	14	14.2	1/10	0.30
5	6.3	Sandy mudstone	10:9:1	63	15.9	1/10	0.21
4	5.8	Coal	10:8:2	58	8.1	1/10	0.09
3	3.0	Sandy mudstone	10:9:1	30	15.9	1/10	0.21
2	7.0	Fine sandstone	7:8:2	70	14.2	1/10	0.30
1	18.4	Sandy mudstone	10:9:1	184	15.9	1/10	0.24

To reduce the influence of boundary effects on the physical simulation, it is generally recommended that the distance between the roadway excavation boundary and the model frame be at least 3–5 times the roadway width. Based on the similarity ratio adopted in this study, the corresponding distance in the model should be approximately 150–250 mm. Therefore, a 300 mm-wide protective coal pillar was reserved between the roadway and the model boundary.

**Fig. 8:**
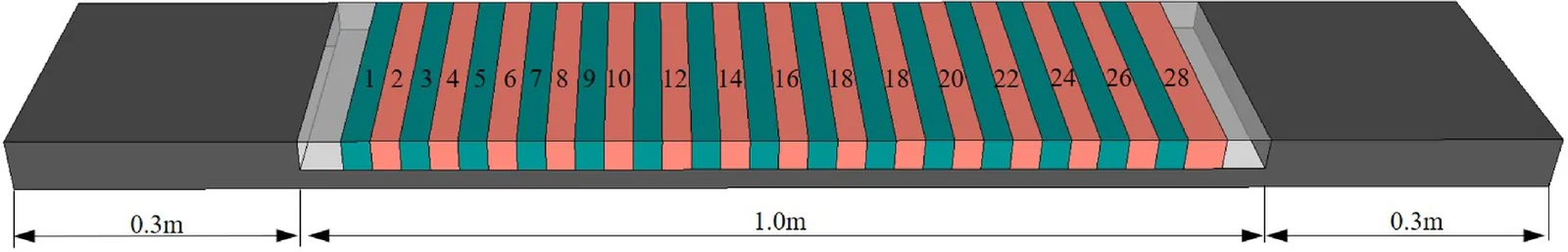
Mining process of coal seam in similar material model.

The mining process was simulated using a cyclic excavation method. The excavation progressed along the strike of the working face at a rate of 6 mm per cut, reaching a total cumulative depth of 400 mm. The excavation was performed sequentially according to the block numbering illustrated in Fig. 8. The process originated from the side of the roof-cut roadway and advanced simultaneously toward the model’s interior and the opposite roadway.

Based on the actual burial depth of the strata, the self-weight stress of the overlying rock mass was converted according to the stress similarity ratio and applied to the top boundary of the model. Consequently, the loading pressure of the top hydraulic actuators was set to 0.26 MPa. According to the in-situ stress measurements, a lateral pressure coefficient of 1.4 was adopted, and the loading pressure applied to the hydraulic actuators on both lateral boundaries was determined to be 0.36 MPa. These boundary loads were maintained until a stable initial in-situ stress field was established within the model.

**Fig. 9:**
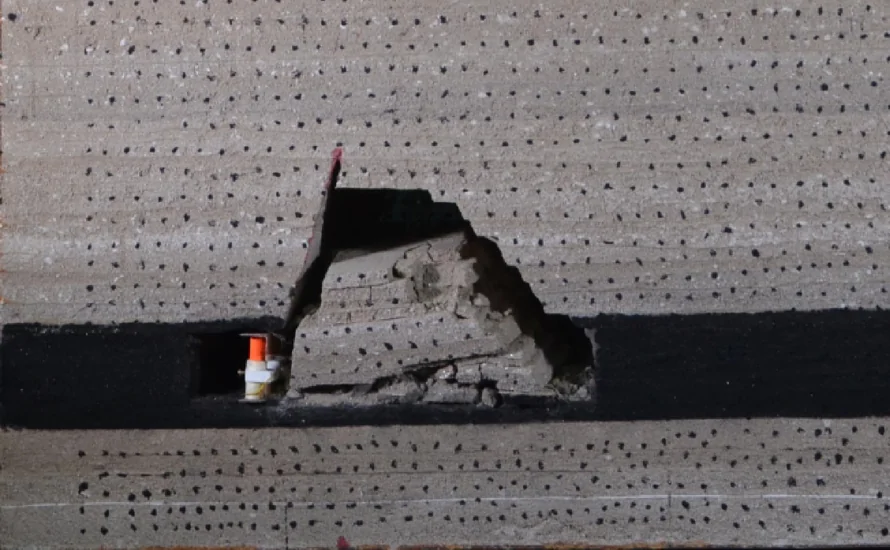
Main roof collapse during the first weighting.

After roadway excavation, mining-induced stress redistribution was simulated through progressive excavation of the working face, thereby reproducing the stress evolution and dynamic loading characteristics associated with panel extraction. The development of roof caving during the first weighting of the main roof is illustrated in Fig. 9.

**Fig. 10:**
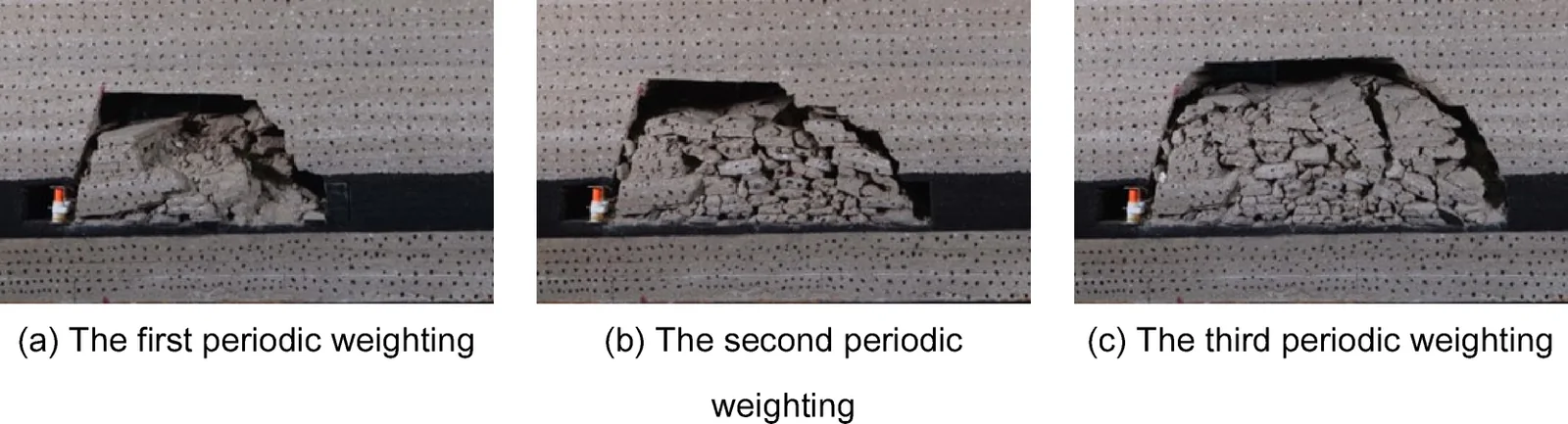
Roof caving during periodic weighting of the main roof.

Subsequently, the working face continued to advance, and the roof underwent three instances of periodic caving. The recorded caving intervals were 12 m, 10 m, and 13 m, respectively, yielding an average periodic weighting interval of 11.7 m. The status of roof caving in the gob during the periodic weighting period is illustrated in Fig. 10.

**Fig. 11:**
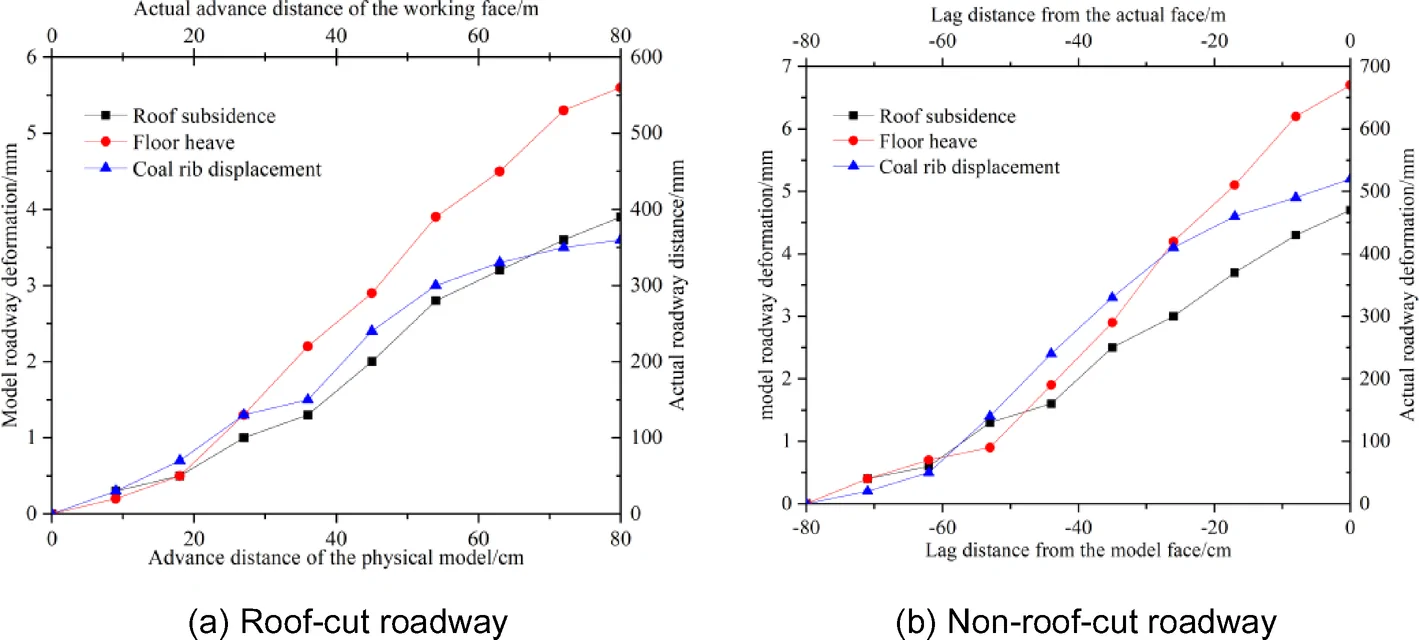
Surrounding rock deformation of roof-cut and non-roof-cut roadways under mining influence.

Displacement monitoring was employed to track the movement of the roadway roof, floor, and solid coal rib during the excavation process. The evolution characteristics of the surrounding rock deformation on both sides of the working face—under both roof-cut and non-roof-cut conditions—were obtained, as illustrated in Fig. 11.

Analysis of the figure reveals that at a lag distance of 80 m behind the working face, the roof subsidence, coal rib deformation, and floor heave in the roof-cut roadway were 390 mm, 350 mm, and 560 mm, respectively. Under identical conditions, the corresponding values for the non-roof-cut roadway were 470 mm, 520 mm, and 670 mm. These results demonstrate that roof cutting exerts a pronounced pressure relief effect on the surrounding rock, thereby effectively mitigating its deformation. Following the implementation of the roof cutting and pressure relief strategy, the roof subsidence, coal rib deformation, and floor heave were reduced by 17%, 48%, and 16%, respectively, leading to significantly enhanced stability of the surrounding rock.

**Fig. 12:**
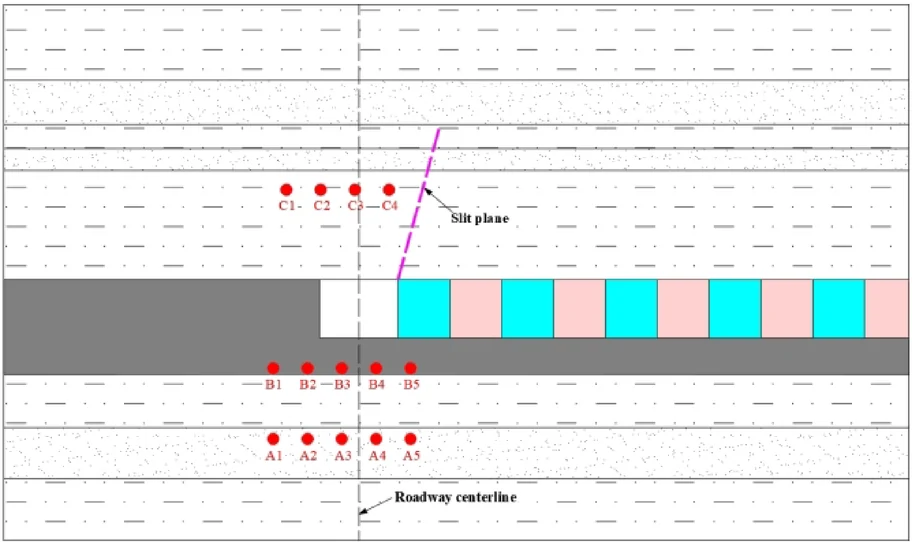
Distribution of strain monitoring points in the surrounding rock of the roof-cut roadway within the model.

Survey lines A, B, and C were selected in the roadway roof and floor for strata strain monitoring. Four strain gauges (C1–C4) were positioned on survey line C, which is located 5 cm above the roof-cut roadway. Along survey lines B and A, situated 2 cm and 5 cm below the floor, a total of 10 strain gauges (B1–B5 and A1–A5) were arranged, respectively. The distances of measuring points C1–C4 from the roof cutting plane were 8 cm, 6 cm, 4 cm, and 2 cm. Measuring points B1–B5 and A1–A5 were offset from the roadway centerline by -5 cm, -3 cm, -1 cm, 1 cm, and 3 cm (where negative values denote positions on the left side of the roadway). The detailed layout of these monitoring points within the model is illustrated in Fig. 12.

**Fig. 13:**
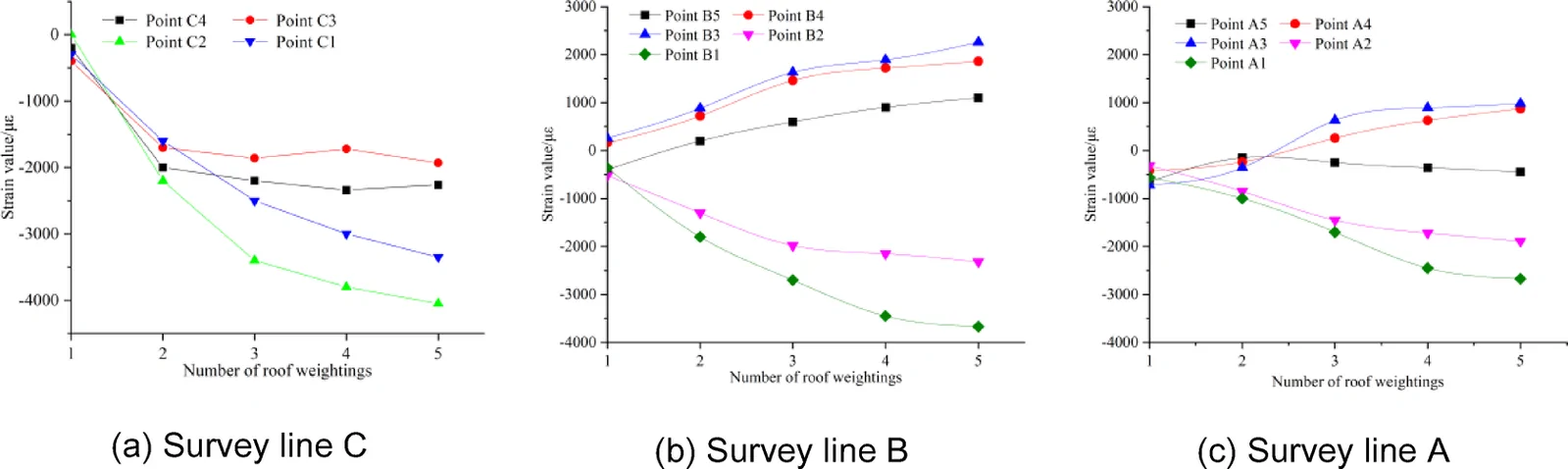
Strain variation curves of the surrounding rock in the roof-cut roadway during mining.

By designating the roof weighting events during the working face extraction as statistical temporal nodes, the strain variation patterns of the gauges embedded within the strata were systematically analyzed. The resulting strain evolution of each measuring point along survey lines A, B, and C is illustrated in Fig. 13.

Analysis of the surrounding rock strain monitoring data reveals the following findings:

1. Under the reinforcement of Constant Resistance and Large Deformation (CRLD) anchor cables and specialized roof-cutting supports, the roof strata of the roof-cut roadway consistently exhibited compressive strain as the working face advanced. The strain in the roadway roof stabilized after reaching a certain threshold. Conversely, the strain in the upper strata of the solid coal rib increased continuously. This disparity is attributed to the effective support provided by the CRLD cables to the roadway roof, whereas the roof above the solid coal side was subjected to intensifying lateral abutment pressure induced by mining.
2. During the advancement of the working face, measuring point B5 (near the floor) transitioned from compressive to tensile strain following coal extraction due to the unloading effect. Meanwhile, points B3 and B4 on the roadway floor remained in a state of tensile strain throughout. This suggests that after the initial weighting, the actual floor failure depth exceeded 2 m. Considering the immediate floor is a 2.3 m coal seam, it is concluded that the floor coal underwent structural failure under the influence of dynamic pressure.
3. Under the continuous influence of periodic weighting, tensile strain was also observed in the sandy mudstone layer situated 5 cm (representing an actual depth of 5 m) below the roadway floor. In contrast, the monitoring points on both sides of the roadway consistently showed compressive strain. These observations indicate that as the extraction area expands, the floor failure depth of the roof-cut roadway can reach up to 5 m.

### Asymmetric floor heave mechanism of gob-side entry retaining by roof cutting under dynamic pressure

Following the mining of the working face, the retained roadway is subjected to mining-induced dynamic pressure. In this study, dynamic pressure mainly refers to the periodic stress disturbance generated by roof weighting, roof fracture and rotation, and the redistribution of abutment pressure. Field monitoring showed that the initial weighting interval was 37 m and the average periodic weighting interval was 10.5 m. During periodic weighting, the hydraulic support load increased from an average value of 20.8 MPa to a peak value of 42.9 MPa, representing an increase of approximately 106%, which reflects the strong dynamic loading characteristics of the overlying strata movement.

Under the repeated action of dynamic pressure, the overburden load is transferred through the roadway roof to the support structures on both sides and subsequently transmitted to the floor strata. Owing to the asymmetric stress distribution between the coal-rib side and gob side, the floor is subjected to uneven compression and progressive plastic deformation. Under the combined effects of horizontal stress and dynamic loading disturbance, the fractured coal floor exhibits squeezing-flow behavior accompanied by tensile cracking and rheological deformation. Therefore, the floor is simplified as a loose granular medium, and the mechanism of asymmetric floor heave is analyzed based on Rankine’s earth pressure theory^27^.

**Fig. 14:**
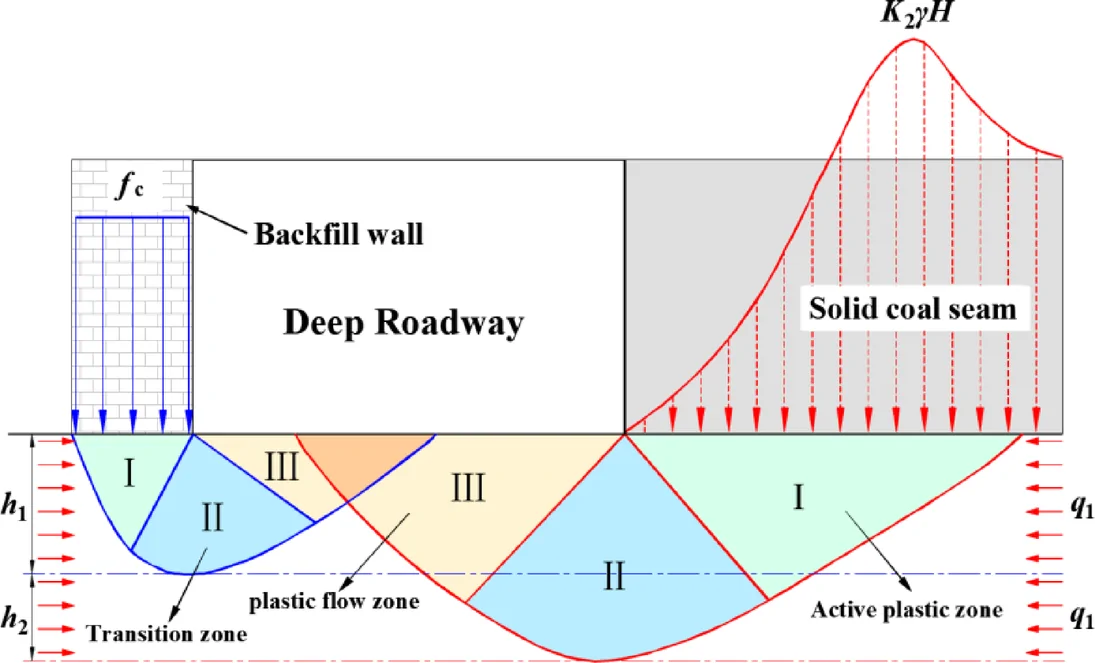
Failure zoning of floor strata in traditional GER under mining influence.

In traditional gob-side entry retaining (GER), an artificial filling wall is typically constructed on one side of the roadway to support the roof. Consequently, the roadside filling wall bears high mining pressure, which is eventually transferred to the underlying floor strata. The loads exerted by these support structures induce plastic deformation in the floor, forming an active plastic deformation zone (Zone Ⅰ). The deformed coal mass then applies horizontal forces to the floor strata; continuous plastic deformation propagates this horizontal action toward the roadway center, where it is finally released through the floor’s free surface. This process creates a transition zone for stress transmission (Zone Ⅱ) and a passive zone where plastic flow deformation occurs (Zone Ⅲ). Due to differences in the spatial position, support width, and mechanical properties of the structures on either side, the size, depth, and distribution of these plastic zones vary significantly. Therefore, floor heave in gob-side entries with roadside filling exhibits pronounced asymmetry. The zoning of floor plastic deformation under mining influence is illustrated in Fig. 14.

In the roof-cutting entry-retaining technology, the traditional roadside filling wall is eliminated and replaced by crushed gangue within the gob to support the overlying strata. During the movement of the overlying strata in the stope, the strength, stress magnitude, and stress distribution characteristics of the gob gangue adjacent to the roadway undergo continuous evolution. To facilitate the application of soil pressure theories in analyzing the stress and deformation patterns of the roadway floor, it is necessary to simplify the mechanical boundary conditions acting upon the floor as follows:

1. It is assumed that under the coupled action of mining-induced pressure, the coal floor transforms into a granular structure with zero cohesion. The equivalent internal friction angle of this granular structure is denoted as *φ*_m_ (°), which is defined by *φ*_m_ = arctan(*σ*_cm_/10), where *σ*_cm_ (MPa) represents the uniaxial compressive strength (UCS) of the coal mass.
2. The force exerted by the gob gangue on the floor coal seam is averaged and simplified as a uniformly distributed load, denoted as *q*_g_ (MPa).
3. In the limit equilibrium state of the roadway floor, the boundary of the active plastic deformation zone furthest from the roadway forms an angle of (45°+*φ*_m_/2) with the horizontal plane, while the boundary of the passive plastic deformation zone forms an angle of (45°-*φ*_m_/2). These two boundaries serve as the interfaces between the deformed and stable regions of the floor strata under the applied load, and are commonly referred to as slip lines.
4. As no aquifers or significant groundwater-bearing formations are developed within the strata surrounding the mined coal seam, the moisture conditions of the floor strata remain relatively stable during mining. Consequently, the influence of water content and its variation on the mechanical behavior of the floor rock mass was assumed to be negligible.

**Fig. 15:**
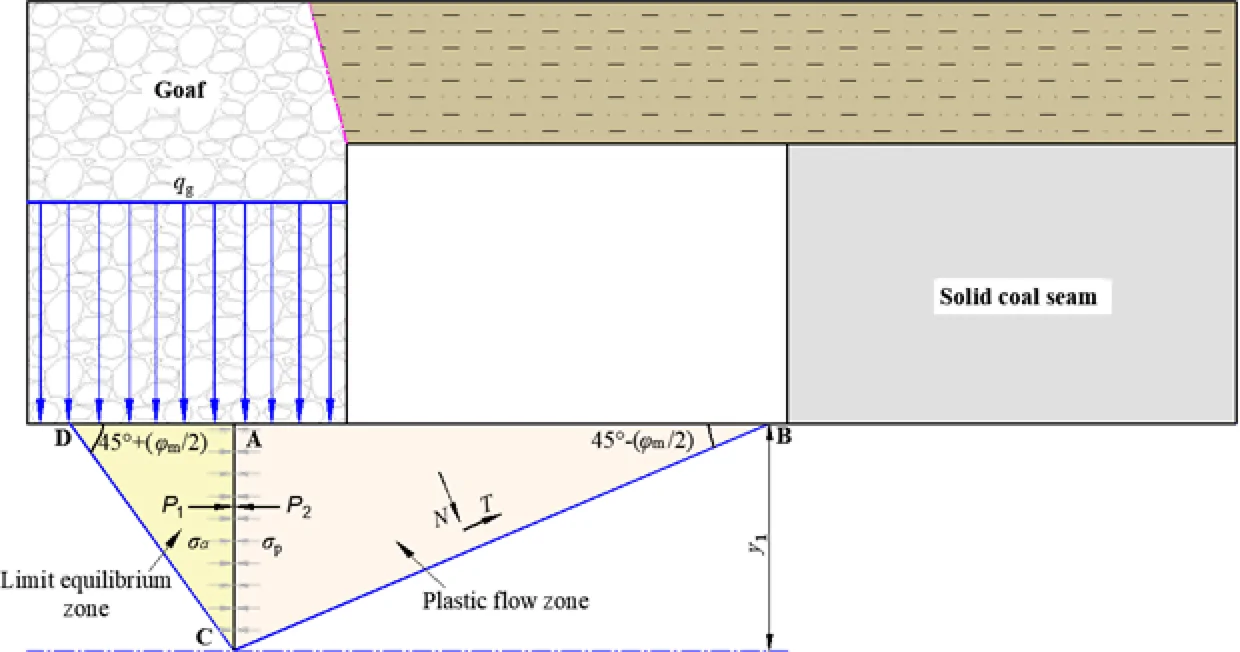
Calculation model for floor failure depth under gangue loading.

Figure 15 illustrates the simplified calculation model for the floor failure depth on the mining side of the roof-cutting roadway.

Under the limit equilibrium state of the roadway floor, zone ACD represents the limit equilibrium zone, while zone ABC denotes the plastic flow deformation zone. Based on Rankine’s earth pressure theory, assuming that line AC acts as a retaining wall, the active earth pressures *σ*_*a*_ (MPa) and passive earth pressures *σ*_*p*_ (MPa) at an arbitrary depth *y* (m) on the retaining wall can be expressed as follows, given the unit weight of the floor strata *γ*_c_ (kN/m^3^) and the active and passive earth pressure coefficients *K*_*a*_ and *K*_*p*_:

1 $$\left\{ {\begin{array}{*{20}l} {\sigma _{a} = \left( {q_{g} + \gamma _{c} y} \right)K_{a} } \hfill \\ {\sigma _{p} = \gamma _{c} yK_{p} } \hfill \\ \end{array} } \right.$$

The active earth pressure coefficient *K*_*a*_ and passive earth pressure coefficient *K*_*p*_ acting on a retaining wall structure are dependent on the internal friction angle *φ*_m_ of the mass. Consequently, the corresponding coefficients for the granular coal mass can be determined using the following equations:

2 $$\left\{ {\begin{array}{*{20}l} {K_{a} = \tan ^{2} \left( {45^\circ - \frac{{\varphi _{m} }}{2}} \right)} \hfill \\ {K_{p} = \tan ^{2} \left( {45^\circ + \frac{{\varphi _{m} }}{2}} \right)} \hfill \\ \end{array} } \right.$$

Zone BCD represents the plastic deformation zone within the roadway floor strata induced by roadside loading, beyond which lies the elastic deformation zone. Consequently, the interface between the elastic and plastic zones is in a critical state. At this juncture, point C on the retaining wall AC reaches a state of limit equilibrium, where the active earth pressure acting on the retaining wall is equal to the passive earth pressure:

3 $$\left( {q_{g} + \gamma _{c} y} \right)K_{a} {\text{ = }}\gamma _{c} yK_{p}$$

Substituting Eq. (2) into Eq. (3) yields the following expression for the failure depth:

4 $$y_{1} = \frac{{q_{g} K_{a} }}{{\gamma _{c} \left( {K_{p} - K_{a} } \right)}}$$

Once the roadway floor heave depth *y*_1_ (m) is determined, the resultant active pressure *P*_1_ (kN) and passive pressure *P*_2_ (kN) acting on the entire retaining wall can be calculated accordingly:

5 $$\left\{ {\begin{array}{*{20}l} {P_{1} = \int_{0}^{{y_{1} }} {K_{a} \left( {q_{g} + \gamma _{c} y} \right)dy} } \hfill \\ {P_{2} = \int_{0}^{{y_{1} }} {\gamma _{c} yK_{p} dy} } \hfill \\ \end{array} } \right.$$

Line BC represents the critical interface of elasto-plastic deformation. Consequently, the difference between the active and passive pressures serves as the primary source of stress driving the relative sliding along this interface. Both the thrust *T* (kN) and the normal force *N* (kN) acting on BC are the components of this active-passive pressure difference resolved along the interface:

6 $$\left\{ {\begin{array}{*{20}l} {T = \left[ {\left( {\frac{1}{2}\gamma _{c} y_{1}^{2} + q_{g} y_{1} } \right)K_{a} - \frac{1}{2}\gamma _{c} y_{1}^{2} K_{p} } \right]\cos \left( {45^\circ - \frac{{\varphi _{m} }}{2}} \right)} \hfill \\ {N = \left[ {\left( {\frac{1}{2}\gamma _{c} y_{1}^{2} + q_{g} y_{1} } \right)K_{a} - \frac{1}{2}\gamma _{c} y_{1}^{2} K_{p} } \right]\sin \left( {45^\circ - \frac{{\varphi _{m} }}{2}} \right)} \hfill \\ \end{array} } \right.$$

By subtracting the frictional resistance Ntan*φ*_m_ acting on the slip surface, the effective thrust *T*_1_ (kN) on the sliding plane is determined as follows:

7 $$T_{1} = \frac{{\left( {\frac{1}{2}\gamma _{c} y_{1}^{2} + q_{g} y_{1} } \right)K_{a} - \frac{1}{2}\gamma _{c} y_{1}^{2} K_{p} }}{{2\cos \left( {45^\circ - \frac{{\varphi _{m} }}{2}} \right)}}$$

The horizontal width *l*_AB_ (m) of the passive plastic deformation zone III, induced by the load exerted by the gangue on the roadway floor, is expressed as:

8 $$l_{{CB}} = \frac{{q_{g} K_{a} }}{{\gamma _{c} \left( {K_{p} - K_{a} } \right)}}\tan \left( {45^\circ - \frac{{\varphi _{m} }}{2}} \right)$$

**Fig. 16:**
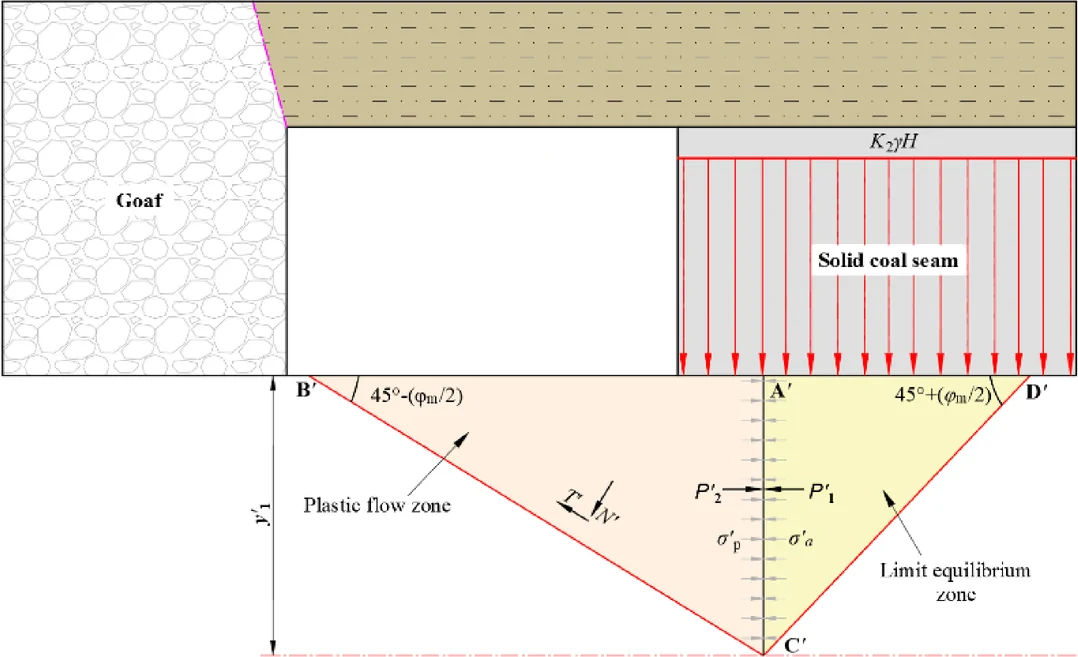
Calculation model for floor failure depth solid coal loading.

Employing the same analytical approach, the force exerted by the solid coal rib on the opposite side of the roadway upon the underlying floor strata is simplified as a uniformly distributed load, *K*_2_*γh*. The resulting simplified calculation model for the floor failure depth on the solid coal side is illustrated in Fig. 16.

The maximum failure depth *y*´_1_ (m) of the floor strata beneath the solid coal rib is given by:

9 $$y_{{_{1} }}^{'} = \frac{{K_{2} \gamma HK_{a} }}{{\gamma _{c} \left( {K_{p} - K_{a} } \right)}}$$

where *K*_2_ is the equivalent lateral abutment pressure coefficient within the solid coal; *γ* is the unit weight of the coal seam, kN/m^3^; *H* is the mining height of the coal seam, m.

The effective thrust *T*´_1_ (kN) along the slip surface B’C’ is expressed as:

10 $$T_{{_{1} }}^{'} = \frac{{\left( {\frac{1}{2}\gamma _{c} y_{1}^{{'2}} + K_{2} \gamma Hy_{{_{1} }}^{'} } \right)K_{a} - \frac{1}{2}\gamma _{c} y_{1}^{{'2}} K_{p} }}{{2\cos \left( {45^\circ - \frac{{\varphi _{m} }}{2}} \right)}}$$

The horizontal width *l*_A´B´_ (m) of the passive plastic deformation zone induced by the load exerted by the solid coal rib on the roadway floor is expressed as:

11 $$l_{{C^{'} B^{'} }} = \frac{{K_{2} \gamma HK_{a} }}{{\gamma _{c} \left( {K_{p} - K_{a} } \right)}}\tan \left( {45^\circ - \frac{{\varphi _{m} }}{2}} \right)$$

As derived from Eq. (4), Eq. (8), Eq. (9), and Eq. (11), the asymmetric load distribution on both sides of the roadway results in non-uniform failure depths and extents within the floor. Furthermore, these failure characteristics undergo continuous evolution in response to the movement of the overlying strata, indicating that the floor heave deformation induced by mining activities exhibits distinct spatio-temporal effects.

**Fig. 17:**
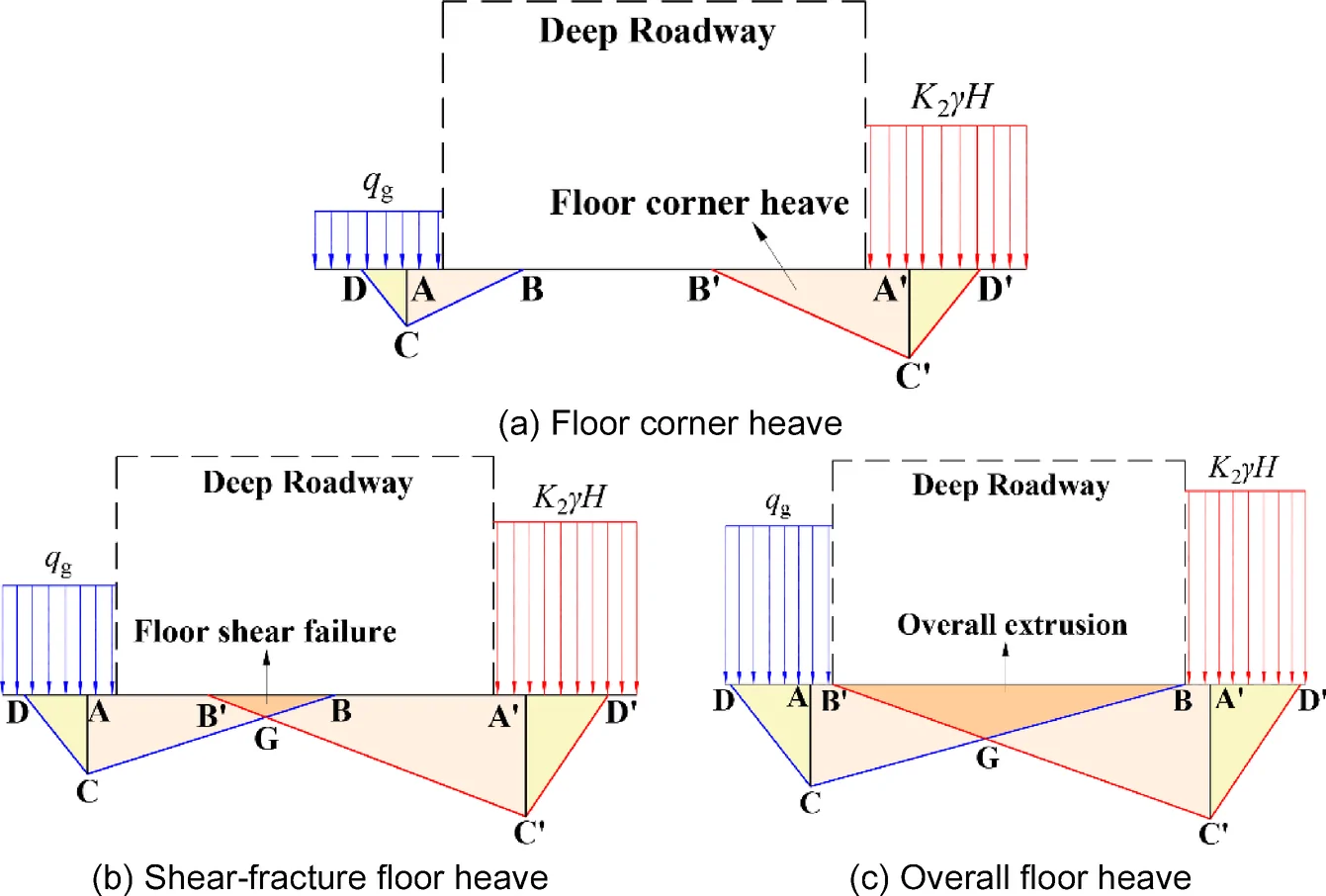
Failure modes of floor heave in roof-cutting & entry-retaining roadways.

1. When the sum of the horizontal widths of the plastic deformation zones (*l*_AB_+*l*_A´B´_) induced by the loads from the solid coal rib and the gob gangue is less than the roadway width *l*_1_, the floor failure zones on both sides of the roadway do not coalesce. Under this condition, floor heave deformation primarily occurs at the roadway sides, manifesting as floor corner heave, as illustrated in Fig. 17a.
2. When the sum of the horizontal widths of the plastic deformation zones (*l*_AB_+*l*_A´B´_), induced by the respective loads from the coal pillar and the gob gangue, exceeds the roadway width *l*_1_ but remains less than twice the roadway width 2*l*_1_, the floor failure zones on both sides of the roadway coalesce. This leads to the formation of block B’GB, resulting in localized floor heave. This phenomenon is categorized as shear-fracture floor heave, as illustrated in Fig. 17b.
3. When the sum of the horizontal widths of the plastic deformation zones (*l*_AB_+*l*_A´B´_), induced by the loads exerted by the bearing structures on both sides of the roadway onto the floor strata, equals twice the roadway width 2*l*_1_, the width of the resulting block B’GB is equal to the roadway width. This state represents the overall floor heave of the roadway, as illustrated in Fig. 17c.

For a gob-side entry retaining roadway formed by roof cutting, the depth of floor failure is governed by the magnitude and distribution range of loads on both sides, as well as the internal friction angle of the floor strata. During the initial stage of entry retaining, the gob gangue is loose and possesses low bearing capacity; consequently, a major portion of the overlying strata load is redistributed toward the solid coal side, resulting in high abutment pressure. At this juncture, the load asymmetry between the two sides of the roadway reaches its peak, manifesting as greater failure depth and more substantial floor heave on the solid coal rib side. As the working face advances, the compaction of the gangue enhances its bearing capacity, thereby mitigating the asymmetry in floor failure and heave, with overall translational characteristics becoming prominent.

In summary, although the roof-cutting and entry-retaining technology is conducive to surrounding rock stability, it increases the width of the limit equilibrium zone within the coal rib. To mitigate this, long anchor cables can be employed to reinforce the solid coal rib prior to entry formation, thereby reducing the width of the limit equilibrium zone. Simultaneously, roadway floor reinforcement measures should be implemented to increase the internal friction angle *φ*_m_ of the floor strata, which effectively minimizes both the depth and the extent of floor failure.

### Multi-level coupled pressure-equalizing control technology for surrounding rock in roof-cutting entry retaining

#### Pre-splitting control technology for roadway roof strata

According to the theory of strata control, the pressure exerted on the roadway roof increases in proportion to the lateral cantilever length of the rock blocks. This constitutes the fundamental mechanical cause for the failure of support structures, the destruction of roadside filling walls, and the significant deformation of the surrounding rock in traditional gob-side entry retaining (GER).

**Fig. 18:**
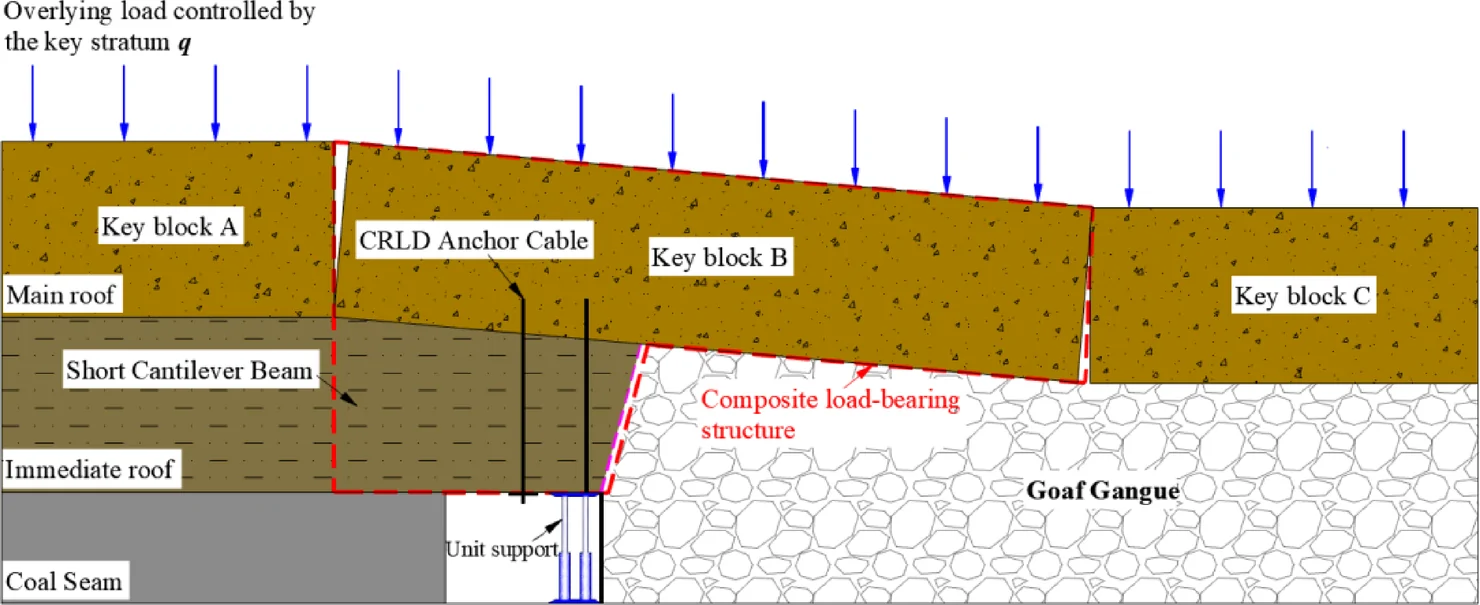
Roof structure model for roof-cutting and entry-retaining roadways.

As the gob roof caving process propagates upward into the overlying strata, the main roof above the coal seam fractures to form an articulated (hinged) rock beam structure. This configuration facilitates the transmission of horizontal stresses through the mechanical interlocking of contact surfaces, thereby creating a “stress arch” effect that sustains the stability of both the structure itself and the underlying rock mass. As illustrated in Fig. 18, key blocks A, B, and C represent the hinged configuration formed above the roadway following the breakage of the main roof along the dip direction of the working face. Specifically, Block A remains relatively stationary with negligible deformation, while Block C, a fractured main roof segment, primarily exhibits translational subsidence. Key block B undergoes rotational subsidence centered on its contact interface with Block A. The coordinated interaction among these three blocks governs the overall stability of the underlying roadway and its surrounding rock.

The rotational subsidence of Key Block B is primarily driven by the overlying load and governed by the gob gangue. By cutting the immediate roof on the gob side of the roadway, the hanging cantilever length of the immediate roof is effectively shortened, resulting in the formation of a “short cantilever beam” structure. Furthermore, roof cutting facilitates the timely caving and bulking expansion of the gob roof, thereby reducing the effective hanging length of the overlying strata. Consequently, the rotation and subsidence of Key Block B are restricted at an earlier stage, leading to a redistribution of the stress field and causing the peak abutment pressure on the coal-rib side to shift deeper into the coal mass.

#### CRLD support technology for the roof and coal rib

**Fig. 19:**
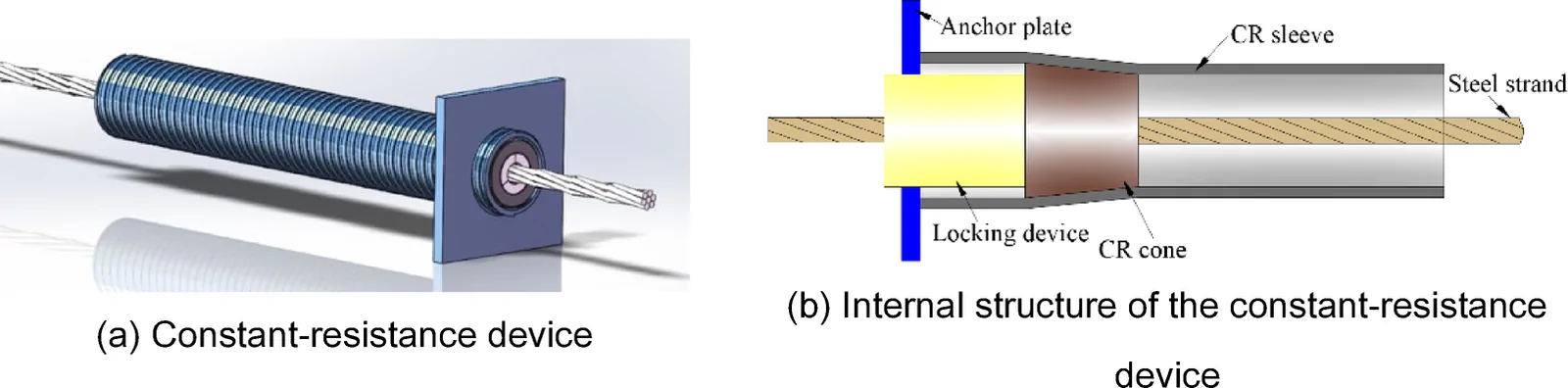
Schematic diagram of the support principle of the constant-resistance device.

Compared with traditional anchor cable support, the mechanical performance of CRLD anchor cables exhibits three distinct advantages: high pre-tension, high constant resistance, and large deformation capacity. These unique mechanical properties are primarily attained through a specialized structural component known as the “constant-resistance device.” As illustrated in Fig. 19, the internal slider within the constant-resistance sleeve slides axially and expands the sleeve upon being tensioned, thereby facilitating energy absorption, yielding, and large deformation of the anchor cable.

**Fig. 20:**
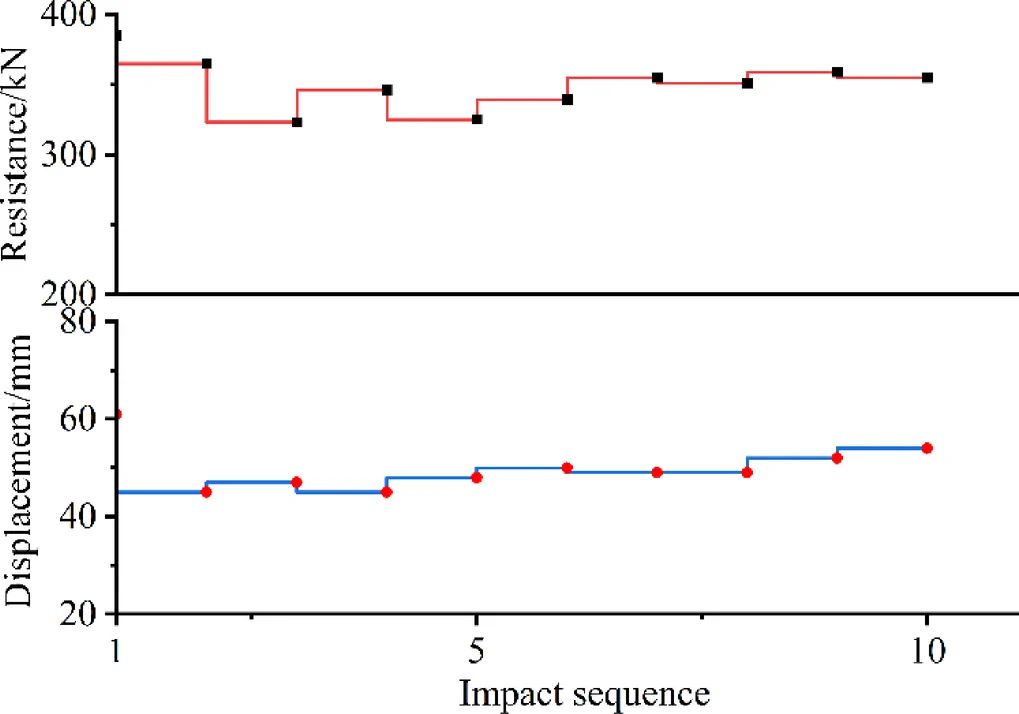
Mechanical properties of CRLD anchor cables under impact loading.

The constant-resistance and yielding characteristics of these anchor cables are of paramount importance for gob-side entry retaining (GER). This is because the primary challenge in surrounding rock control within GER stems from the influence of mining-induced pressure. By employing laboratory impact load tests to simulate the dynamic pressure triggered by roof fracturing during the mining process, it was observed that the working resistance of the CRLD anchor cable was consistently maintained at approximately 350 kN throughout 10 consecutive impacts. Furthermore, the total deformation exceeded 400 mm, demonstrating its superior performance in maintaining surrounding rock stability under dynamic loading. These test results are illustrated in Fig. 20.

With the implementation of CRLD anchor cable support, the immediate roof strata above the roadway can be suspended to Key Block B. The high pre-tension and constant-resistance yielding mechanical properties of these anchor cables ensure that the immediate roof and the main roof function as an integrated unit. Simultaneously, the overall integrity of the coal rib is enhanced under the reinforcement of the CRLD anchor cables, which optimizes the stress distribution within the coal pillar. Finally, the bulked gangue in the gob gradually recovers its stress-bearing capacity and inherent strength through the process of sustained compression.

### Pressure-equalizing support technology using inverted floor beams for deep roadways under dynamic loading

Based on the aforementioned investigation into the floor heave mechanism of coal seam roadways, the critical factor in controlling floor heave within roof-cutting roadways under dynamic pressure lies in suppressing asymmetric deformation while simultaneously enhancing the equivalent internal friction angle *φ*_m_ of the floor strata. Squeeze-flow floor heave should be mitigated through floor support or reinforcement measures; specifically, shear-fracture floor heave triggered by non-uniform support strength must be averted. Grounded in these insights, this study proposes an inverted floor beam pressure-equalizing support technology for roof-cutting roadways under dynamic loading.

**Fig. 21:**
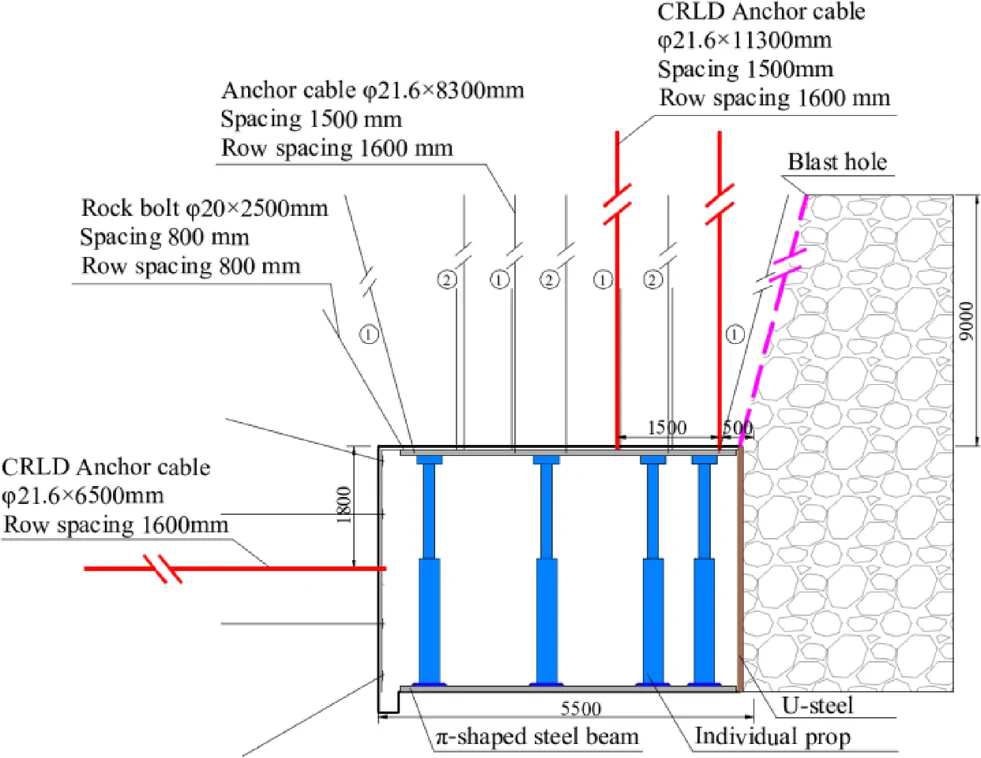
Pressure-equalizing support scheme using inverted floor beams for roof-cutting and entry-retaining roadways.

The roadway roof is reinforced with Φ21.6 × 11,300 mm CRLD anchor cables, while the coal rib is supported by Φ21.6 × 6500 mm CRLD anchor cables for reinforcement. For temporary internal support, a “two-beam and four-column” configuration is adopted, consisting of π-shaped steel beams positioned at the roof and floor along with four individual hydraulic props. The comprehensive support scheme for the roadway and the surrounding rock is illustrated in Fig. 21.

The application of inverted floor beams facilitates the transfer of roof pressure to the floor beam, which subsequently transmits a quasi-uniformly distributed support pressure to the coal floor through the contact interface. This mechanism realizes pressure-equalizing support for the floor strata, thereby enhancing their overall integrity and increasing the equivalent internal friction angle *φ*_m_. Concurrently, the reinforcement of the solid coal rib with CRLD anchor cables serves to contract the width of the limit equilibrium zone within the rib. Consequently, this mitigates the depth of the floor failure zone induced by the concentrated loads exerted from the coal rib side.

To verify the floor control efficacy of the pressure-equalizing support technology using inverted floor beams, two alternative schemes were proposed for comparison. The rationality and effectiveness of the inverted floor beam support technology were subsequently evaluated through a comparative analysis of field-measured deformation data.

**Fig. 22:**
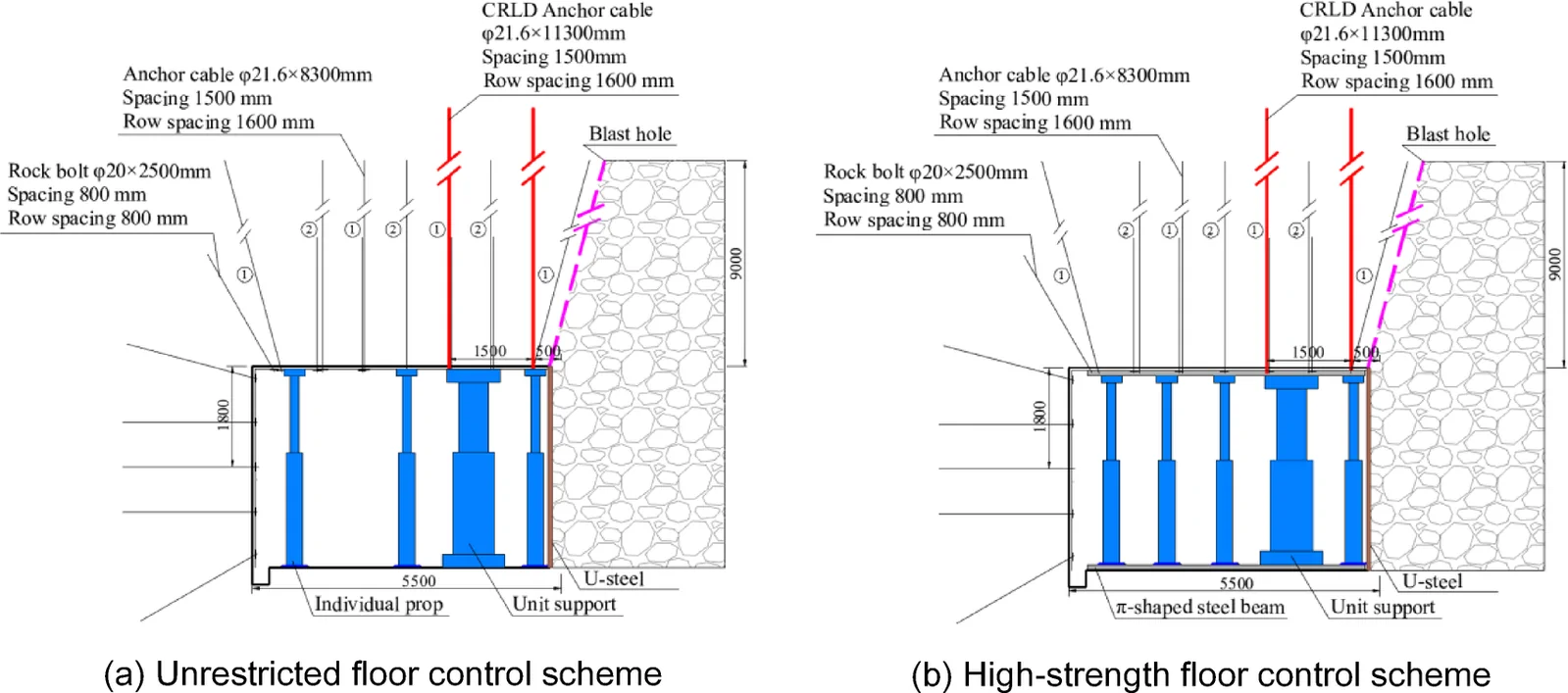
Two comparison schemes of internal roadway support for floor heave control.

Comparative Scheme 1: Unrestricted floor control scheme (Fig. 22a). In this scheme, only individual hydraulic props and unit supports are employed for roof and floor support, allowing the roadway floor to fully release its deformation energy. This configuration is designed to measure the actual magnitude of floor heave in the roof-cutting roadway under the influence of dynamic pressure at the 16,011 working face of the Zhaogu No. 1 Mine.

Comparative Scheme 2: High-strength floor control scheme (Fig. 22b). In this configuration, a combination of “unit supports + two-beam and four-column” is adopted for temporary internal support. Specifically, the unit supports and one individual hydraulic prop are positioned near the gangue side of the roadway to provide sufficient roadside support resistance for the “cantilever beam” structure. Notably, the coal rib in this scheme is not reinforced with CRLD anchor cables.

### Engineering application

**Fig. 23:**
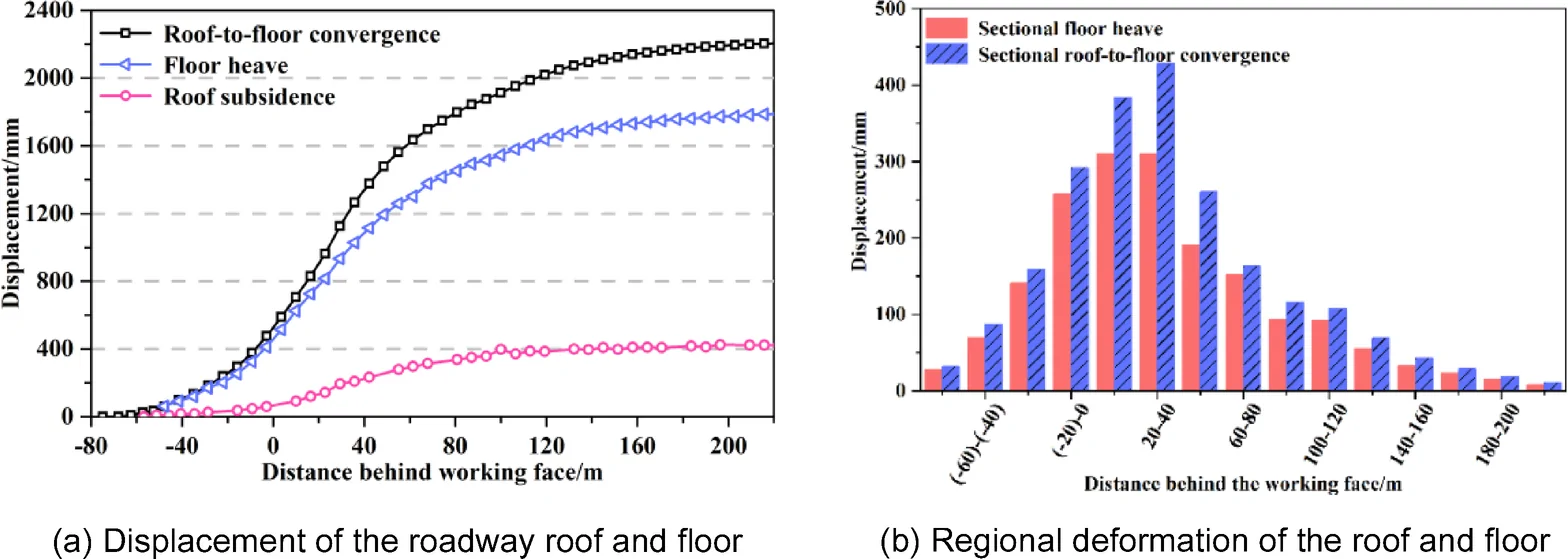
Surrounding rock deformation of the roadway under the unrestricted floor control scheme.

During the application of the roof-cutting and entry-retaining technology, the surrounding rock deformation in the roadway section where Comparative Scheme 1 was implemented was continuously monitored. The resulting evolution law of the surrounding rock deformation is illustrated in Fig. 23.

As shown in Fig. 23a, in the absence of targeted control measures for the roadway floor, the relative roof-to-floor convergence exceeds 2,200 mm, with floor heave accounting for 81.1% of the total deformation. Analysis of Fig. 23b reveals that the interval from 40 m ahead of the working face to 80 m behind it represents a phase of rapid floor heave development, during which 82.1% of the total floor heave magnitude occurs. It is evident that the primary influence zone of dynamic mining pressure extends from 40 m in front of the working face to 80 m behind it, with the resulting deformation being predominantly characterized by floor heave.

**Fig. 24:**
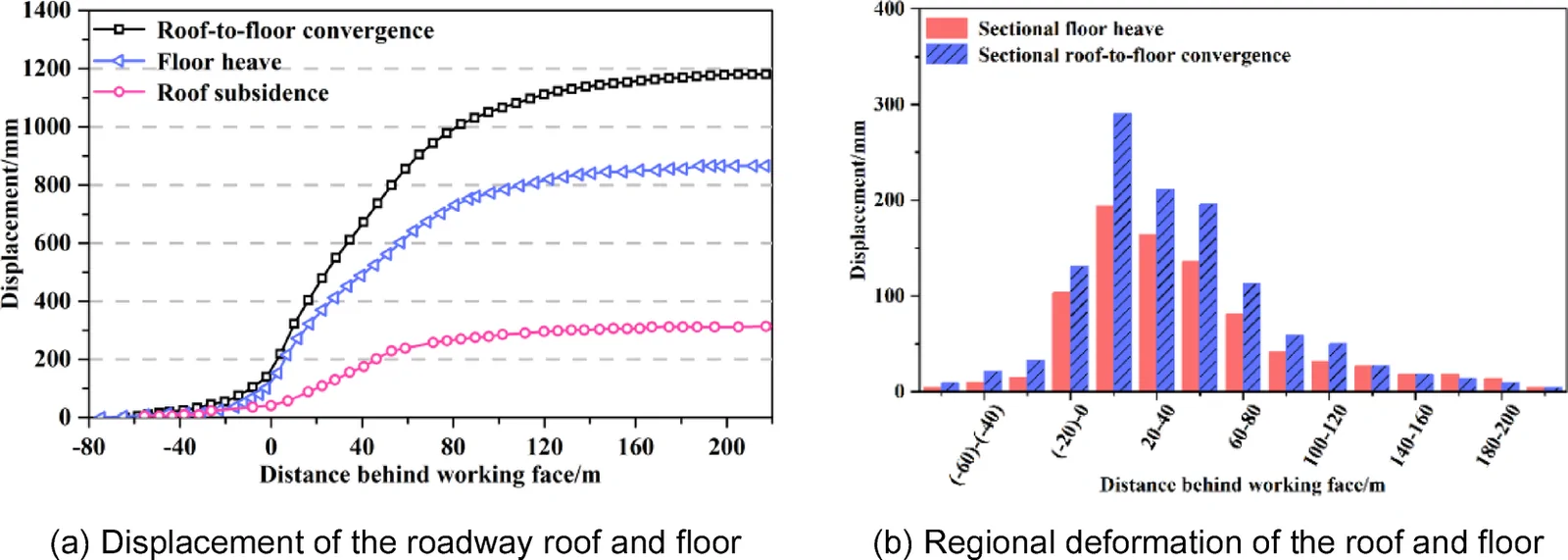
Surrounding rock deformation of the roadway under the high-strength floor control scheme.

Following the implementation of the high-strength floor control scheme (Comparative Scheme 2), the monitored evolution law of the surrounding rock deformation is illustrated in Fig. 24.

As shown in Fig. 24a, after increasing the internal temporary support strength and incorporating the inverted floor beam, the roof-to-floor convergence of the roadway is 1,179 mm, with floor heave accounting for 72.9% of the total deformation. Analysis of Fig. 24b indicates that the interval from 20 m ahead of the working face to 80 m behind it constitutes the phase of rapid floor heave development, during which 78.7% of the total floor heave magnitude occurs.

**Fig. 25:**
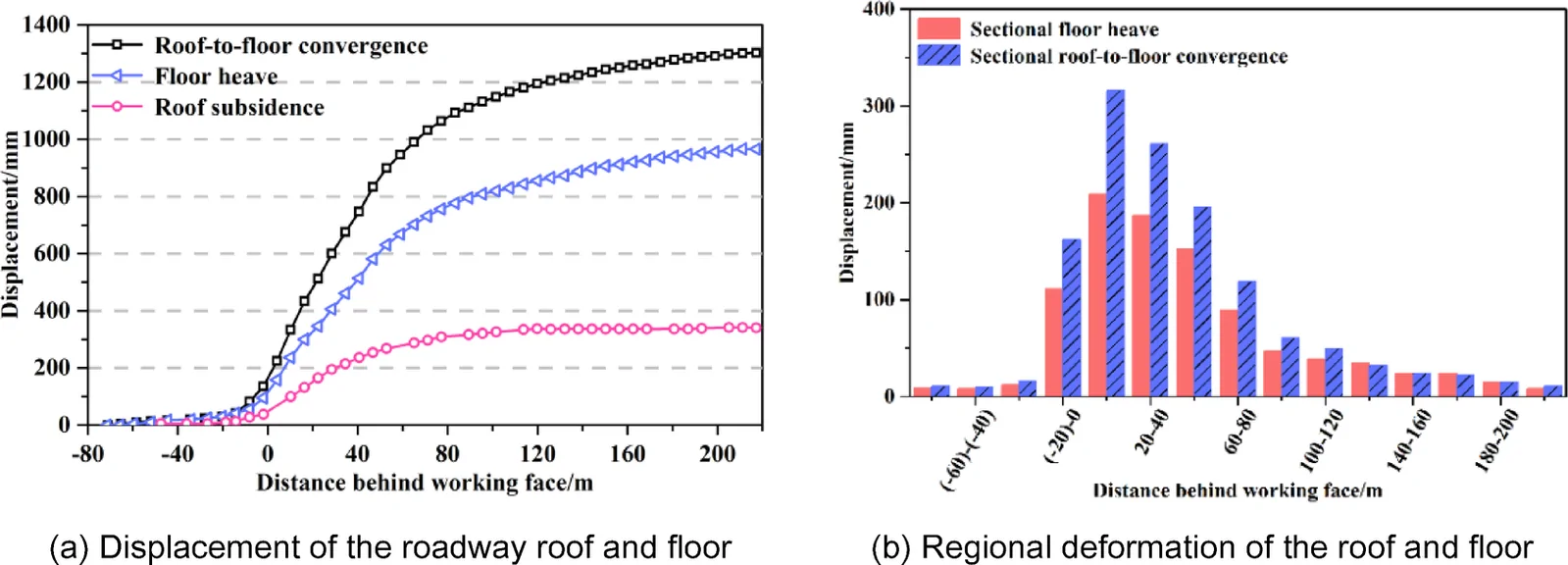
Surrounding rock deformation of the roadway under the pressure-equalizing support.

Following the implementation of the pressure-equalizing support scheme using inverted floor beams, the monitored evolution law of the surrounding rock deformation is illustrated in Fig. 25.

As shown in Fig. 25a, after implementing the roof-rib-floor coupling support technology, the roof-to-floor convergence of the roadway is 1,301.9 mm, with floor heave accounting for 74.1% of the total deformation. Analysis of Fig. 25b indicates that the interval from 20 m ahead of the working face to 80 m behind it represents the phase of rapid floor heave development, during which 77.2% of the total floor heave magnitude occurs.

Comparing the three options indicates that the floor heave reached 1787 mm in the unrestricted floor control scheme. This deformation was significantly suppressed to 866 mm (a 51.5% decrease) by the high-strength floor control scheme, and to 956 mm (a 46.5% decrease) by the pressure-equalizing support scheme.

The deformation mechanism and evolution process of asymmetric floor heave under dynamic pressure were analyzed using theoretical calculations and physical similarity simulations. The field geological and mining parameters were substituted into the proposed equations to calculate the floor failure depth and plastic zone width. Furthermore, borehole camera inspections were carried out to obtain the corresponding field measurements. The comparison results presented in Table 3 show that the maximum deviation between the theoretical predictions, physical simulation results, and field observations is less than 10%. This good consistency demonstrates the reliability of the proposed mechanical model and provides further support for the inferred mechanism of asymmetric floor failure.

**Table 3:** Comparison of asymmetric floor failure characteristics obtained from theoretical analysis, physical simulation, and field monitoring.

Characteristic	Physical similarity simulation	Theoretical calculations	Field observations
Dominant failure location	Coal-rib side floor	Coal-rib side floor	Coal-rib side floor
Failure depth (m)	5.4	5.1	5.5
Plastic zone width (m)	4.5	4.5	4.5
Floor heave mode	Asymmetric squeezing-flow	Asymmetric squeezing-flow	Asymmetric squeezing-flow
Crack propagation pattern	Shallow → deep	Shallow → deep	Consistent
Deformation concentration zone	Coal-rib side	Coal-rib side	Coal-rib side

A synthesis of the aforementioned deformation monitoring data yields the following findings: by enhancing the lateral support strength of the coal rib with CRLD anchor cables, and implementing floor pressure-equalizing control through the combination of single hydraulic props and inverted floor beams, the resulting floor heave is slightly greater than that of the high-strength floor control scheme. However, this scheme eliminates the need for unit supports and simultaneously reduces the consumption of individual hydraulic props by 25%, thereby saving support investment and maintenance workload. Consequently, for deep roof-cutting and entry-retaining roadway with coal floors, the pressure-equalizing support scheme is optimal alternative.

## Conclusion

- The asymmetric movement laws of overlying strata in roof-cutting and entry-retaining roadways were investigated through a physical similarity model test conducted in deep soft rock. Monitoring results demonstrate that, compared to the non-roof-cutting roadway, the roof subsidence, solid coal rib deformation, and floor heave of the entry after roof cutting and pressure relief were reduced by 17%, 48%, and 16%, respectively.
- Subject to dynamic loading, the roadway floor experiences progressive tensile strain as the working face advances, which subsequently induces tensile failure. As the failure depth continuously increases, floor heave gradually emerges as the predominant component of surrounding rock deformation. When the distance lagging behind the working face exceeds 70 m, the floor failure depth surpasses 5 m.
- A Rankine-based mechanical model for asymmetric floor failure under roof-cutting gob-side entry retaining conditions was established. Theoretical formulas for the floor failure depth and the width of the passive plastic deformation zone—considering the loading effects of both the solid coal rib and crushed gangue—were derived respectively. Consequently, the failure evolution mode of the floor in roof-cutting roadway was identified. Mechanical analysis further indicated that floor heave can be effectively controlled by enhancing the lateral support strength of the coal rib and increasing the equivalent internal friction angle *φ*_m_ of the floor strata.
- A coupled support strategy integrating roof pre-splitting, CRLD anchor cable reinforcement, and inverted floor beam support was proposed for asymmetric floor heave of the 16,011 tailentry. This scheme aims to cut off stress transfer toward the retained roadway, reinforce the coal rib, and achieve pressure equalization within the floor through the inverted floor beam. This integrated roof–rib–floor support system promotes the recovery of the overall strength and stability of the surrounding rock mass, effectively mitigating the asymmetric floor heave and deformation caused by dynamic pressure disturbances during mining. Field comparative tests demonstrate that, compared to the unrestricted floor control scheme, the proposed scheme reduced floor heave deformation by 46.5%.

This study systematically investigated the mechanism and control of asymmetric floor heave under mining-induced dynamic pressure in roof-cutting gob-side entry retaining through theoretical analysis, physical similarity simulation, and field validation. The proposed roof–rib–floor collaborative support scheme effectively mitigated severe asymmetric floor deformation. Future research should focus on integrating Digital Image Correlation (DIC) for full-field deformation monitoring, investigating the long-term creep behavior of floor strata under sustained dynamic loading, and evaluating the applicability of the proposed support strategy under high-moisture and groundwater-rich conditions.

## Data Availability

The data used to support the findings of this study are available from the corresponding author upon reasonable request.
